# A review of materials used in tomographic volumetric additive manufacturing

**DOI:** 10.1557/s43579-023-00447-x

**Published:** 2023-08-29

**Authors:** Jorge Madrid-Wolff, Joseph Toombs, Riccardo Rizzo, Paulina Nuñez Bernal, Dominique Porcincula, Rebecca Walton, Bin Wang, Frederik Kotz-Helmer, Yi Yang, David Kaplan, Yu Shrike Zhang, Marcy Zenobi-Wong, Robert R. McLeod, Bastian Rapp, Johanna Schwartz, Maxim Shusteff, Hayden Talyor, Riccardo Levato, Christophe Moser

**Affiliations:** 1 Ecole Polytechnique Féderale de Lausanne, 1015 Lausanne, Switzerland; 2 Department of Mechanical Engineering, University of California, Berkeley, CA USA; 3 John A. Paulson School of Engineering and Applied Sciences, Harvard University, Cambridge, MA USA; 4 Wyss Institute for Biologically Inspired Engineering, Harvard University, Boston, MA USA; 5 Department of Orthopaedics, University Medical Center Utrecht, Utrecht University, Utrecht, The Netherlands; 6 Lawrence Livermore National Laboratory, Livermore, CA USA; 7 Department of Mechanical Engineering, Technical University of Denmark, 2800 Kongens Lyngby, Denmark; 8 Institute of Microstructure Technology (IMTEK), University of Freiburg, Georges Köhler Allee 103, 79110 Freiburg, Germany; 9 Department of Chemistry, Technical University of Denmark (DTU), 2800 Kongens Lyngby, Denmark; 10 Center for Energy Resources Engineering (CERE), Technical University of Denmark (DTU), 2800 Kongens Lyngby, Denmark; 11 Department of Biomedical Engineering, Tufts University, Medford, MA 02155 USA; 12 Division of Engineering Medicine, Department of Medicine, Harvard Medical School, Brigham and Women’s Hospital, Cambridge, MA 02139 USA; 13 Tissue Engineering + Biofabrication Laboratory, Department of Health Sciences & Technology, ETH Zürich, Otto-Stern-Weg 7, 8093 Zurich, Switzerland; 14 Materials Science and Engineering Program, University of Colorado, Boulder, USA; 15 Department of Electrical, Computer and Energy Engineering, University of Colorado, Boulder, USA; 16 Department of Clinical Sciences, Utrecht University, Utrecht, The Netherlands

## Abstract

Volumetric additive manufacturing is a novel fabrication method allowing rapid, freeform, layer-less 3D printing. Analogous to computer tomography (CT), the method projects dynamic light patterns into a rotating vat of photosensitive resin. These light patterns build up a three-dimensional energy dose within the photosensitive resin, solidifying the volume of the desired object within seconds. Departing from established sequential fabrication methods like stereolithography or digital light printing, volumetric additive manufacturing offers new opportunities for the materials that can be used for printing. These include viscous acrylates and elastomers, epoxies (and orthogonal epoxy-acrylate formulations with spatially controlled stiffness) formulations, tunable stiffness thiol-enes and shape memory foams, polymer derived ceramics, silica-nanocomposite based glass, and gelatin-based hydrogels for cell-laden biofabrication. Here we review these materials, highlight the challenges to adapt them to volumetric additive manufacturing, and discuss the perspectives they present.

**Graphical abstract:**

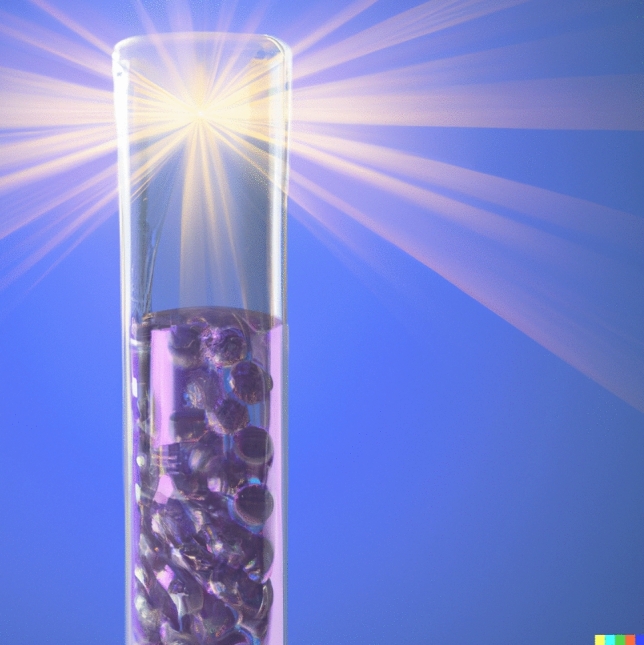

**Supplementary Information:**

The online version contains supplementary material available at10.1557/s43579-023-00447-x.

## Introduction

**Figure 1:**
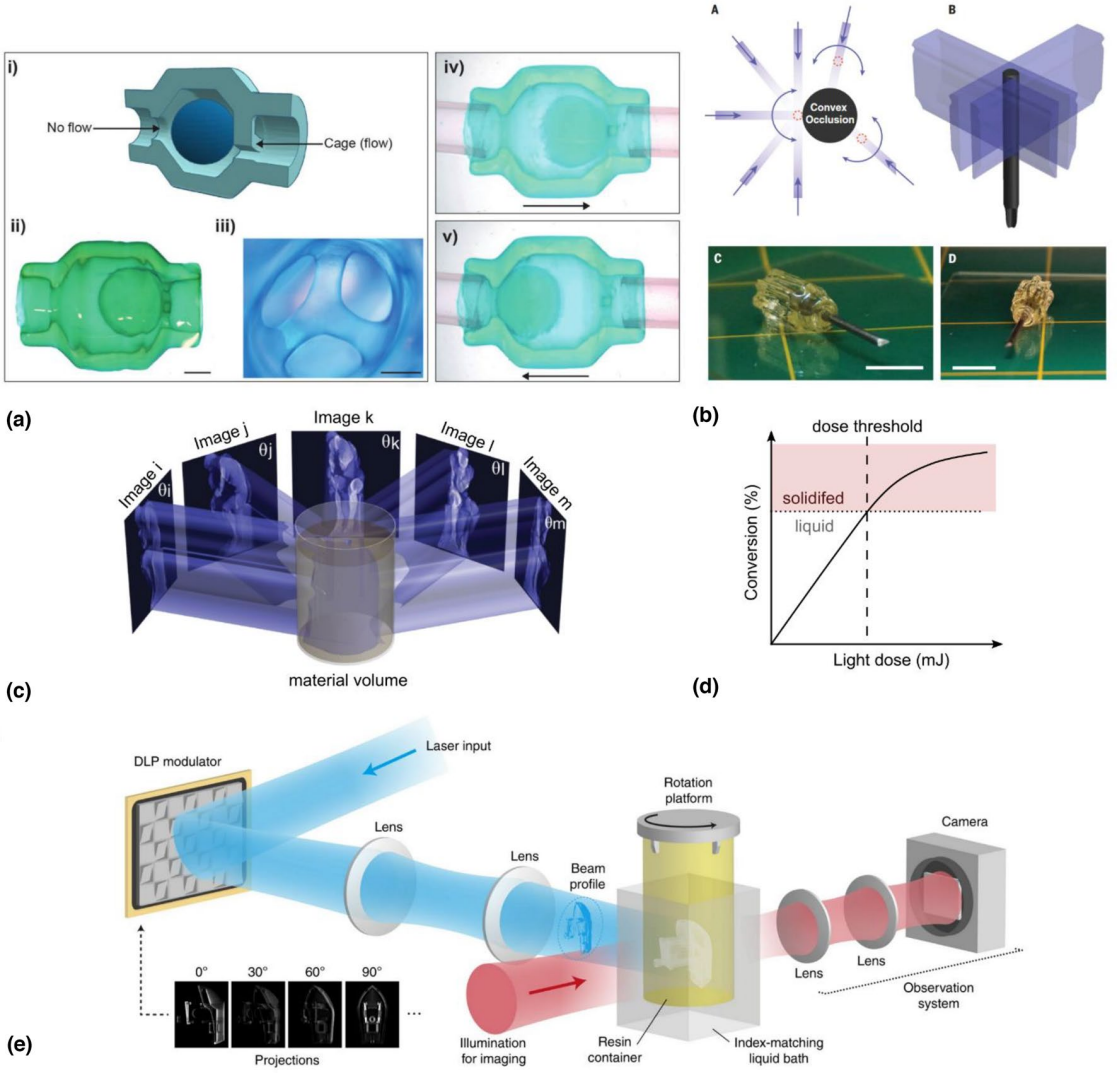
(a) Design freedom of tomographic volumetric additive manufacturing (VAM) is demonstrated by the fabrication of a fluidic ball-cage valve with free-floating elements (scale bars = 1 mm, Copyright Wiley) and (b) by the overprinting of an acrylic screwdriver handle around its metallic shaft (scale bars = 10 mm, Copyright AAS. (c). In VAM, an entire three-dimensional object is simultaneously solidified by irradiating a liquid photopolymer volume from multiple angles with dynamic light patterns (Copyright AAAS). (d) VAM exploits the thresholded response of corresponding photosensitive materials to light-induced polymerization. Thanks to this solidification threshold, only the target object is printed, even if the resin outside the object’s target volume inevitably receives some light after having been illuminated from multiple angles. The liquid unpolymerized resin can be washed away after the print. (Copyright De Gruyter). (e) Exemplary setup for VAM (Copyright Springer-Nature).

3D printing has revolutionized the manufacturing industry by simplifying the fabrication of designs with complex geometries. Light-based 3D printing exploits the ability of certain light-sensitive molecules to trigger polymerization or crosslinking reactions in liquid resins, thus solidifying them. Different ways of bringing light to the print have led to the development of stereolithography (SLA),^[1]^ Digital Light Processing (DLP),^[2,3]^ selective laser sintering (SLS),^[4]^ and more recently two photon polymerization (2PP),^[5,6]^ xolography,^[7]^ light sheet microprinting,^[8]^ and tomographic volumetric additive manufacturing or computed axial lithography.^[9,10]^ In many of these methods, support struts are needed for complex designs with overhangs or cavities. In others, the object is printed within the resin itself, which supports it. Volumetric methods, such as two photon polymerization, and more recently xolography and tomographic volumetric additive manufacturing offer full design freedom. Fig. 1(a) and (b) illustrates design freedom with a fluidic ball-cage valve with free-floating elements and a screwdriver handle overprinted around a metallic shaft, both printed through tomographic volumetric additive manufacturing (VAM). In VAM, an entire three-dimensional object is simultaneously solidified by irradiating a volume of liquid photocurable resin from multiple angles with dynamic light patterns [Fig. 1(c)].^[9,10]^ Unlike most other additive manufacturing methods, tomographic volumetric additive manufacturing is layerless, meaning that it does not fabricate objects by solidifying one voxel, one line, or one layer at the time. Instead, light from the subsequent tomographic patterns builds up an energy dose within the complete volume of the target object. Typical printing times are in the order of tens of seconds^[10]^ for cm-scale prints with resolutions down to 50 to 80 μm.^[11,12]^ Additionally, the technique has proven versatile and has been used to fabricate objects in materials such as acrylates, thiol-enes,^[13–15]^ nanoparticle-loaded composites,^[11]^ polymer-derived ceramics,^[16]^ epoxies,^[17]^ silk bioinks,^[18]^ and cell-laden hydrogels.^[10,19–23]^

In this review, we will cover the underlying functioning mechanism of VAM, briefly discuss the optical and computational strategies developed so far to improve fidelity and resolution, cover in detail its applicability to a large variety of materials (Table I), and present our perspective on future applications, challenges and possibilities.

**Table I:** Formulations used for volumetric additive manufacturing.

Type	Formulation	Photoinitiator (concentration)	Wavelength	Typical viscosity	Other components	Notes
Acrylate^[12,34]^	Di-pentaerythritol pentaacrylate	TPO (0.6 mM)	405 nm	> 10 Pa·s	TiO2 was added into the resin in one of these works to make it scattering	
Silicone^[12]^	93 wt% vinyl-terminated PDMS + 4.7 wt% fumed silica reinforced vinyl-terminated PDMS + 2.3 wt% (mercaptopropyl) methylsiloxane–dimethylsiloxane	TPO-L (2.25 mM)	405 nm			Demonstration of 3D printing of full silicones
Acrylate^[9]^	BPAGDA 75 wt% + PEGDA 25 wt%	CQ (5.2 mM) + EDAB 1:1 (weight ratio)		5.2 Pa·s		
Acrylate^[48]^	Urethane Dimethacrylate	2-Benzyl-2-dimethylamino-1-(4-morpholinophenyl)-butanone-1 (6 mM)	405 nm	11 Pa·s		Printed in microgravity
Acrylate^[48]^	Aliphatic urethane acrylate diluted in isobornyl acrylate (EBECRYL 242 N)	2-Benzyl-2-dimethylamino-1-(4-morpholinophenyl)-butanone-1 (6 mM)	405	25 Pa·s		Printed in microgravity
Acrylate—epoxy ^[17]^	PEGDA / BPAGDA / EEC	CQ (0.02 wt%) EDAB (0.02 wt%) CAT2 (2.9 wt%)	455 / 365 nm			CAT2 first dissolved in propylene carbonate
Acrylate ^[17,31]^	PEGDA / BPAGDA	CQ (0.1 wt%) EDAB (0.25 wt%)	455 nm	0.32 Pa·s		
Acrylate^[31]^	PEGDA / BPAGDA	CQ (0.1 wt%) EDAB (0.25 wt%)	455 nm	0.32 Pa·s	TEMPO (0.004 or 0.01 wt%)	
Acrylate^[17,31]^	PEGDA / BPAGDA	CQ (0.1 wt%) EDAB (0.25 wt%)	455 nm	1.40 Pa·s		Viscosity adjusted adding BPAGDA
Acrylate^[30,31]^	TEGDMA / BisGMA	CQ (0.2 wt%) EDAB (0.5 wt%)	460/365 nm		*o-*Cl-HABI (1 or 3 wt%)	HABI pre-dissolved in THF
Acrylate^[30,31]^	TEGDMA / BisGMA	CQ (0.1 wt%) EDAB (0.25 wt%)	460/365 nm		*o-*Cl-HABI (0.4 wt%)	HABI pre-dissolved in THF
Polymer-derived ceramic^[16]^	Polysiloxane (SPR 684) 85 wt% + 1,4-butanediol diacrylate 15 wt%	TPO (2 mM, 0.063 wt %)	405 nm	0.87 Pa·s		
Nanocomposite for glass^[11]^	296 g/mol trimethylolpropane triacrylate (TMPTA) + hydroxyethylmethacrylate(HEMA)	CQ (0.117 wt%) + EDAB (0.117 wt%)	442 nm	10 Pa·s	TEMPO (0.2 M) in TMPTA (0.5 vol%)	
Shape memory foam^[14]^	TEGDAE: TA-ICN: TME-ICN (0.1: 0.9: 1 molar equivalent of functional groups)	2-Methyl-4′-(methylthio)-2-morpholinopropiophenone (10 mM)	405 nm		23.5 mM ANPHA + 0.1 mM TEMPO	
Thiol-ene^[13]^	TEGDA + TEGDAE + TAE-ICN + TA-ICN + TME-ICN (in different mixing molar ratios)	2-Methyl-4′-(methylthio)-2-morpholinopropiophenone (10 mM)	405 nm		TEMPO 0.1 mM	
Poly(ε-caprolactone)^[15]^	PCL-ene/ PETA-4SH	TPO-L (0.12–0.25% w/v)	442 nm		TEMPO (0.1 mg mL-1)	
Hydrogel (cell-laden)^[10]^	GelMA 10% w/v	LAP (0.037% wt)	405 nm	Thermally gelated		Printing time ≈12.5 s, viability > 85%
Hydrogel (organoid-laden)^[20]^	GelMA 5% w/v	LAP (0.1% w/v)	405 nm	Thermally gelated	Iodixanol	Iodixanol concentration needs to be optimized for each material and cell type. Laden with up to 5 million cells mL^−1^
Hydrogel (cell-laden)^[19]^	GelNB/PEG4SH 2.5% w/v	LAP (0.05% w/v)	405 nm	Thermally gelated		Laden with murine C2C12 myoblasts at 1 million cells mL^−1^
Hydrogel (cell-laden)^[23]^	GelMA 5, 8 and 15% w/v	LAP (0.1% w/v)	405 nm	Thermally gelated		Performed in presence of melt electrowritten poly(ε-caprolactone) meshes
Hydrogel (cell-laden)^[45]^	GelMA 10% w/v in PBS	LAP (0.16 mg mL^−1^)	405 nm	Thermally gelated		Laden with human fibroblasts at 0.5 million cells mL^−1^
Hydrogel^[18]^	Silk sericin 2.5–5% w/v	Ru/SPS (1/10) (0.25–1 mM Ru)	525 nm	Not measured	Water	Printing time ~ 55–80 s C2C12 myoblasts at 5 million cells mL^−1^
Hydrogel^[18]^	Silk fibroin 1.25–15% w/v	Ru/SPS (1/10) at (0.125–1 mM Ru)	525 nm	Not measured	Water	Printing time ~ 30–170 s
Hydrogel^[22]^	GelNB/PEG4SH	LAP (0.05% w/v)	405 nm	Thermally gelated		Printing time ≈10–11 s, viability > 95%
Hydrogel^[22]^	GelNB/GelSH	LAP (0.05% w/v)	405 nm	Thermally gelated		
Hydrogel^[22]^	PVA-NB/PEG2SH	LAP (1.7–2 mM)	405 nm	Thermally gelated	Sacrificial Gelatin	

## 3D printing as tomographic back-projection

A series of 2D light patterns are projected from many different angles onto the vial of photocurable resin to build a cumulative light dose matching the geometry of the printed object [Fig. 1(c)]. Although the entire volume will be irradiated with light, only the portion where light dose is sufficiently high to surpass a solidification threshold is solidified [Fig. 1(d)]. The projected patterns correspond to the tomographic projections of the desired object, and they can be calculated using the Radon transform or the Fourier slice theorem.^[24,25]^ Because cartesian 2D images are projected onto a rotating vial (which is better described by polar or cylindrical coordinates), some frequencies get more highly sampled than others, and the resulting projections are blurred.^[26]^ To correct for this, projected patterns can be filtered in the frequency domain, as is the case in filtered back-projection algorithms.^[27]^ However; filtered back-projections include negative values. Projecting negative light intensities is physically possible but technically challenging. It may be done through interference^[28]^ or by producing photoinhibition instead of photoinitiation.^[29–31]^ So far, however, most works have employed simpler approaches in which negative values are set to zero following a non-negativity constraint. Iterative algorithms can be applied to the resulting physically-incorrect patterns to improve print fidelity.^[9,32,33]^

### Optomechanical setup

The optomechanical setup required for tomographic volumetric printing consists of a light source, a light modulator with a projection system, and a rotational stage for the photocurable resin [Fig. 1(e)]. The light source can be either a laser^[10–12,34]^ or a LED^[9,13,17,18]^ with a wavelength matching the absorbance spectrum of the used photo initiator. High-power LED are less expensive than laser sources, but their larger *étendue* makes them more divergent and compromises resolution.^[12]^ 3D ray tracing can be applied to compensate for optical aberrations from divergence and lack of telecentricity.^[35]^ The light modulator typically consists of a digital micromirror device that displays the calculated patterns synchronously with the rotation of the vial. Some works have also used commercial beamers as projectors.^[36]^

Printing occurs within sealed transparent cylindrical vials, unlike open vats common to DLP or SLA. This reduces contamination risks in bioprinting, for example, as well as it contains fumes when volatile resins are used. However, cylindrical vials induce lensing and aberrations, which can be corrected by using a refractive-index matching bath^[12]^ or through software-based corrections of the illumination patterns.^[37]^ Additionally, imaging systems can be added to the setup to monitor or control the progression of printing. These systems typically use red light to avoid inducing undesired photopolymerization. Boniface et al*.* included a motorized linear stage to have the resin follow a helical motion in the printer, enabling the fabrication of larger objects still in a layer-less manner.^[38]^

### Resolution, print fidelity & smoothness: advanced printing strategies

**Figure 2:**
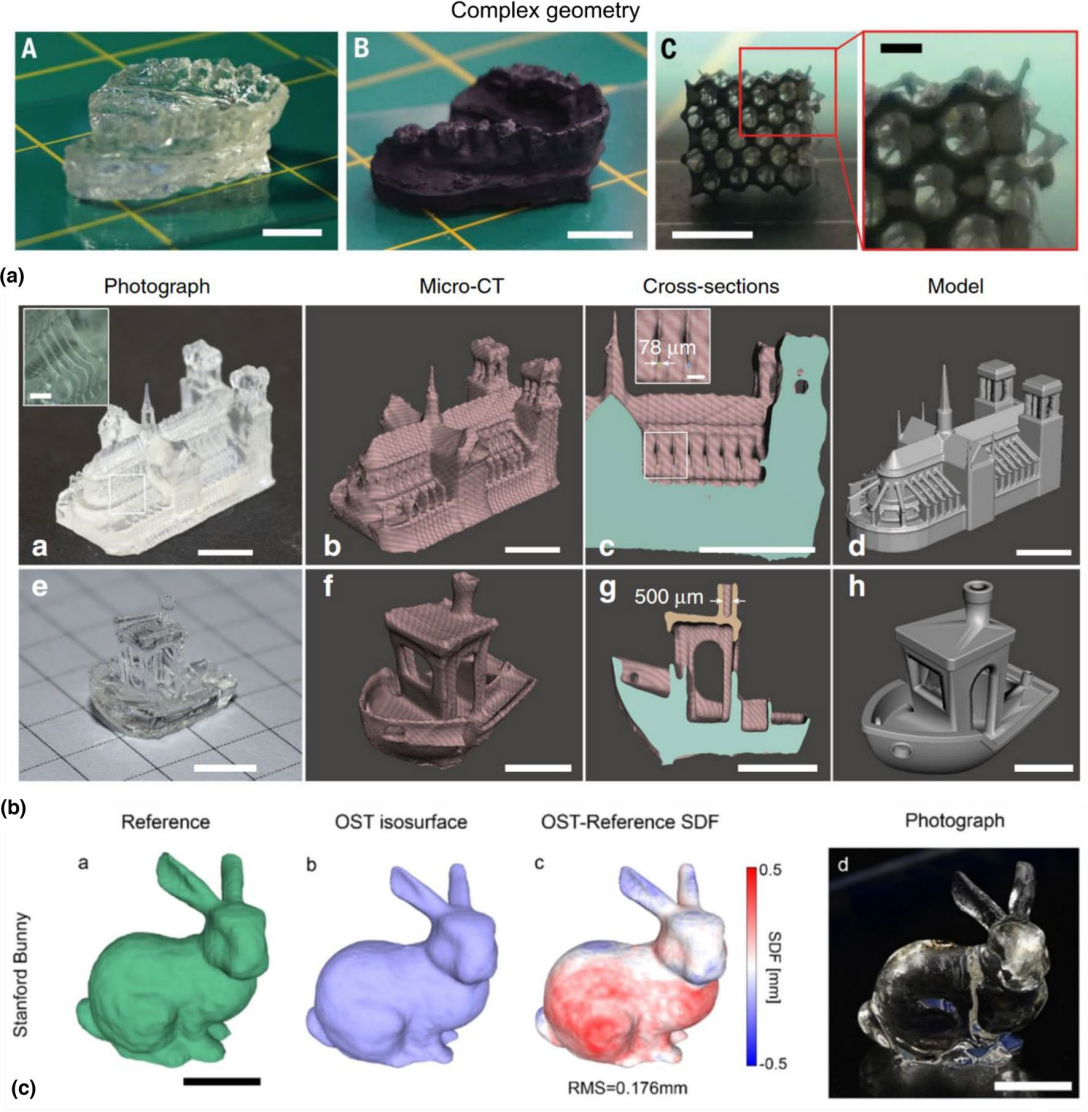
(a) VAM allows for the rapid fabrication of complex geometries into acrylates (scale bars = A.A-B 10 mm, A.C 5 mm, A.D 2 mm; copyright AAAS).^[9]^ (b) High resolution of positive and negative features is achievable using sacrificial prints (scalebars 5 mm, insets (ba) 1mm, (bc). 0.5 mm; Copyright Springer-Nature).^[12]^ (c) Optical scattering tomography can be used to determine automatically when to stop the print to maximize fidelity (scale bars = 5 mm; Copyright Elsevier).^[41]^

VAM exhibits unprecedent printing speed and enormous versatility across materials. However, the achievable resolution is still limited to above 50 µm. Different strategies to increase print fidelity and resolution have been presented and include adaptations to the calculations of the projected patterns,^[32,34,39]^ optical corrections to reduce aberrations,^[35,37]^ and feedback from sacrificial prints^[12,40]^ or live feedback to stop the excitation light [Fig. 2(b) and (c)].^[41]^ Moreover, refractive-index changes induced by photopolymerization can produce lensing artifacts, including striations via self-writing waveguides.^[42]^ Such striations degrade print shape accuracy and give VAM-printed parts layer-like effects despite VAM being free from layering. Rackson et al. presented an ingenious strategy to mitigate striations and produce smooth shapes in VAM by flooding the vat with uniform light the end of the printing process.^[43]^

The smallest printable feature size is at best limited by the projected image of the DMD micromirrors. Beam divergence further decreases resolution, which is why low-étendue light sources (such as laser diodes) are preferable than high-étendue sources (such as LEDs). This means that resolution can’t be any better than the size of the DMD micromirror on the image plane. As an example, Toombs et al. used lower magnification in their micro-CAL setup to demonstrate the fabrication of 3D objects with minimal feature sizes of 20 and 50 μm in polymer and fused silica glass, respectively; albeit at the cost of smaller printable sizes.^[11]^ The minimal fabricated features were much larger than the projected DMD mirror images in this work. Resolution is further limited due to materials, light deposition, and tomographic calculations, among others (Tables II and III).

**Table II:** Fabrication methods combined with VAM and their demonstrated applications.

Combined fabrication method	Materials	Description
Extrusion bioprinting^[171]^	Cell-laden jammed microgels (extrusion) + cell-laden GelMA (VAM)	microgels laden with iβ pancreatic cells were extruded into a GelMA hydrogel laden with human mesenchymal stem cells. The surrounding GelMA hydrogel is shaped by VBP
Melt electrowriting^[23]^	Poly-ε-caprolactone (MEW) + cell-laden GelMA hydrogels (VBP)	Two-step VBP in human mesenchymal stem cells around a pre-fabricated melt-electrowritten poly-ε-caprolactone mesh. The poly-ε-caprolactone enhances the mechanical properties of the construct, which can be ultimately seeded with human umbilical vein endothelial cells to produce vein models
Photopatterning^[22,170]^	GelNB (VAM) + growthfactors (photopatterning); PVA hydrogel + fluorescent dyes	GelNB is photocrosslinked into a desired shape by VBP, then growth factors are diffused into the gel and photopatterned onto a desired 3D geometry also through tomographic light projections
Sequential multimaterial VAM ^[9,23,145]^	Acrylates around metals; GelNB (different formulations); cell-laden GelMA (different cell types)	Overprinting around prefabricated part
Two-photon abalation^[144]^	GelNB	VBP is used to fabricate acellular constructs with hollow cavities (400 μm). Then, microcavities (down to 2 µm) are fabricated into the gel by two-photon ablation

**Table III:** Abbreviations used throughout this text.

Abbreviation	Definition	Section	Notes
ANPHA	Aluminum N-nitrosophenylhydroxylamine	Thiol-enes	Material (Pot-life stabilizer)
BisGMA	Bisphenol A-glycidyl methacrylate		Material
BPAGDA	Bisphenol A glycerolate (1 glycerol/phenol) diacrylate		Material
CAT2	Triarylsulfonium hexafluoroantimonate salts	Epoxies	Material
CQ	Camphorquinone		Photoinitiator
DIW	Direct ink writing	Sinterable materials	Fabrication method
DLP	Digital light printing		Fabrication method
EDAB	Ethyl 4-(dimethylamino)benzoate	Epoxies	Photoinitiator
EEC	3,4-Epoxycyclohexylmethyl 3,4-epoxycyclohexanecarboxylate		Material
FTIR	Fourier transform infrared	Thiol-enes	Analysis method
GelMA	Gelatin methacryloyl	Hydrogels	Material (hydrogel)
GelNB	Gelatin-norbornene	Hydrogels	Material (hydrogel)
HEMA	Hydroxyethylmethacrylate		Material
LAP	Lithium phenyl-2,4,6-trimethylbenzoylphosphinate	Hydrogels	Photoinitiator
*o*-CL HABI	2.2′-Bis (2-chlorophenyl)-4,4′,5,5′-tetraphenyl1,1′-biimidazole; 1H-Imidazole, 2-(2-chlorophenyl)-1-[2-(2-chlorophenyl)-4,5-diphenyl-2H-imidazol-2-yl]-4,5-diphenyl	Epoxies	Photoinitiator
PCL	Poly(ε-caprolactone)	Thiol-enes	Material
PEG	Polyethylene Glycol	Hydrogels	Material
PEGDA	Polyethylene Glycol Diacrylate		Material
PVA	Polyvinyl alcohol	Hydrogels	Material
Ru/SPS	Ruthenium/sodium persulfate	Hydrogels	Photoinitiator
SF	Silk fibroin	Hydrogels	Material (protein)
SLA	Stereolithography		Fabrication method
SMP	Shape Memory Polymers		Family of materials
SS	Silk sericin	Hydrogels	Material (protein)
TA-ICN	Tri-allyl isocyanurate	Shape memory foams	Material
TAE-ICN	Tris[2-(acryloyloxy)ethyl] isocyanurate	Thiol-enes	Material
TEGDA	Triethylene glycol diacrylate	Thiol-enes	Material
TEGDAE	Triethylene glycol diallyl ether	Shape memory foams	Material
TEGDMA	Triethylene glycol dimethacrylate		Material
TEMPO	2,2,6,6-tetramethyl-1-piperidinyloxy		Material (nitroxyl radical)
THF	Tetrahydrofuran	Epoxies	Material
TMPTA	Trimethylolpropane triacrylate		Material
TME-ICN	(Tris[2-(3-mercaptopropionyloxy)ethyl] isocyanurate	Shape memory foams	Material
TPO	Diphenyl(2,4,6-trimethylbenzoyl)phosphine oxide		Photoinitiator
TPO-L	2,4,6-Trimethylbenzoyldi-Phenylphosphinate (liquid)	Thiol-enes	Photoinitiator
VAM	Volumetric additive manufacturing	Introduction	Fabrication method
VBP	Volumetric BioPrinting. Equivalent to VAM, when applied to the fabrication of cell- or organoid-laden contructs for tissue engineering	Hydrogels	Fabrication method

Chemical diffusion of free radicals (be it from the activated photoinitiator or growing polymer chains) also reduces resolution. Radical quenchers, such as TEMPO, can be used to limit the detrimental effects of radical diffusion and dark curing.^[44]^ In addition to this, limited light contrast also hinders resolution.

As light patterns traverse the entire vial’s volume, there is light deposited in regions outside of the target volume. Algorithms that optimize light patterns so less light goes outside of the build volume can improved print fidelity.^[39]^

Fabricating objects with sub-wavelength features with a purely back-projection approach will be challenging. However, integrating two-photon or two-step absorption into the fabrication process may bridge this resolution gap.

### Resin viscosity

Because objects are not fabricated in a layer-by-layer fashion, the resin does not need to flow at each printing step, as is the case in SLA or DLP; more viscous resins can be used in VAM. This has enabled the use of solvent-free formulations, which have higher monomer concentrations and thus polymerize faster and yield stronger objects, and has also enabled the use as solid or gelled materials, like hydrogels^[10,18–22,34,45]^ and organogels.^[46]^ Part sedimentation during the printing process depends on print shape and resin viscosity and could compromise print fidelity if not taken into account properly.^[47]^ Previous works have shown that sedimentation dos not hinder fidelity in viscous resins, as it occurs mostly once printing has finished.[12, p. Supp. Mats.],[16, p. Supp. Mats.] On the contrary, when the polymerization is highly exothermic, solidified objects can float instead of sinking in VAM. Fabricating objects under microgravity opens the possibility to use less viscous or more exothermic materials.^[48]^ Viscosity, however, is not a fundamental requirement for VAM, and printing in low-viscosity materials could be done by reducing the amount of time between the beginning of solidification and the end of the print, or by adjusting the displayed patterns to the expected sedimentation.

### Optical transparency

As VAM relies on light exciting the full volume at once and not layer by layer, high optical transparency is a requirement for the usable materials. Because of this, photoinitiators must be used at low concentrations, otherwise light would be rapidly attenuated following the exponential decay described by Beer–Lambert–Bouguer law. Ideal photoinitiators for VAM have low extinction molar coefficients but high polymerization yield. As seen in Table I, BAPO, TPO, and camphorquinone are commonly used initiators in VAM, having molar extinction coefficients in the order of 100–200 L mol^−1^ cm^−1^ at the excitation wavelength;^[49]^ which is orders of magnitude lower than the peak molar extinction coefficients other photoinitiators. Their low concentration is, however, extremely beneficial in some applications such as in bioprinting given the cytotoxicity of most photoinitiators. Light absorbers and dyes, which are common in DLP and SLA, are actually detrimental to VAM because they limit the penetration depth of light.

Many resin formulations of interest are scattering, such as cell-laden hydrogels or composite resins. Scattering deviates light from the straight path that it is assumed to follow in the computations for the tomographic patterns. The detrimental effect of scattering has been mitigated by reducing the refractive index mismatch within the components of the resin^[11,20]^ or by including the scattering profile of the material in the computational pipeline.^[34]^ Possibly, scattering could be reduced by using longer-wavelength photoinitiators,^[50]^ upconversion nanoparticles,^[51,52]^ or multi-photon instead of single-photon excitation.^[5,53,54]^

Polymerization may be induced with radiation outside of the visible spectrum, such as with microwaves. Although inherently subject to lower resolution due to the wave nature of light, tomographic microwave curing could be used to fabricate objects volumetrically in completely opaque materials.^[55]^ Acoustic waves could also be used holographically or tomographically to fabricate 3D objects in opaque media.^[56]^

## Materials used for VAM

### Acrylates

VAM was first introduced with acrylated urethanes thanks to their high reactivity, low cost, and availability in commercial coatings, adhesives, and vat 3D printing applications.^[9,12]^ They are also highly transparent, even in the near UV, and have tunable mechanical properties. Polyacrylates, having multiple functional domains, polymerize rapidly and in a propagating reaction initiated by a free radical from photoinitiators.^[57]^ This polymerization reaction terminates spontaneously when two chains bind or when molecular oxygen, in dilution in the resin, scavenges free radicals.^[57,58]^ Oxygen-mediated inhibition gives a non-linear thresholded conversion response of acrylates to light dose, which facilitates printing in VAM [Fig. 1(d)].^[59]^

Objects printed in acrylates are typically stiff enough to retain their shape, but soft enough to open up new possibilities for elastomers, challenging materials to 3D print but with numerous industrial applications, like personalized hearing aids^[12]^ or dental retainers^[9,38]^ [Fig. 2(a)]. Also, Rodríguez-Pombo et al*.* loaded polyethylene glycol diacrylate (PEGDA) hydrogels with paracetamol to simultaneously produce several tablets through VAM within seconds,^[60]^ opening possibilities for personalized tablet manufacturing, although challenges remain to make the photo-crosslinkable hydrogel edible.

### Epoxies and orthogonal polymerization

3D printers that can not only shape devices but also modulate their mechanical properties spatially enable applications in emerging fields like soft robots and electronics.^[61,62]^ Two strategies are employed to generate property gradients within a single workpiece: (i) greyscale printing^[63]^ and (ii) orthogonal polymerization.^[64]^ In greyscale printing, property modulation relies on the spatial accumulation of single-wavelength light dose, in combination with a well-defined correlation relating dose and extent of reaction to a property in question. The achievable property gradient reflects an overlap between the photoresponse of the polymer precursor and the established dose contrast within the process window (i.e. within the printing time). For example, illumination can be modulated to change the glass transition temperature above or below room temperature, leaving the end product in a rubbery or glassy state, to further increase the achievable contrast.^[63]^ Dose modulation in TVAM is challenging: the highest dose is capped by the need to minimize over-exposed out-of-part voxels, while the lowest dose is defined by the gelation threshold. The short printing time of VAM limits the achievable hardening, whereas undesired dose accumulation due to full-body illumination limits retainable softness. Nonetheless, stiffness modulation via grayscale printing is achievable using smart algorithm design,^[17,39]^ or by reversing dose buildup through pseudo-negative illumination.^[30,31]^

Introducing orthogonal mechanisms of photo-polymerization adds another dimension to property modulation. Free-radical mediated chain growth is advantageous in VAM because the curing threshold set by pre-dissolved radical quenchers is essential for establishing a desired dose contrast that guarantees the geometric fidelity of reconstruction, and the termination of propagation is amenable to actinic control. In contrast, cationic polymerization does not offer a threshold-setting mechanism that separates in-part voxels from out-of-part voxels, and the sustained chain growth, even in the dark, poses additional challenges for arresting conversion and for post-processing. The idea of selectively curing a resin into different materials with exposure to different light sources in a one-vat setup was first explored by Decker,^[65]^ and later by Ruiter et al*.*^[66]^ In the latter work, a monomer is functionalized with an oxetane and an acrylate group, and the authors selectively polymerized only the acrylate with a radical photoinitiator (PI) using visible light. The coexisting epoxy monomer was only initiated with a cationic PI when exposed to light of wavelength below 385 nm. Larsen et al*.*,^[64]^ using a mixture of hydrogel and epoxide precursors, showed that blue light could excite the free radical PI without initiating the epoxy group, whereas the opposite is not true—upon excitation by UV, a cationic PI produces both a Brønsted acid that initiates epoxy, and free radical species that initiate acrylate. As a result, two polymers are interlaced but not covalently bonded, allowing for greyscale printing to control the extent of both reactions separately. The cited formulation can generate a continuous variation in mechanical property, ranging from stiff thermoset to much softer hydrogel-like materials. Schwartz and Boydston demonstrated promising applications of orthogonal polymerization in 3D- and 4D-printing using a DLP-based, dual-wavelength setup.^[67]^ Schmidleithner adopted the strategy to enhance the interfacing between microfluidic devices and the peripheral.^[68]^

**Figure 3:**
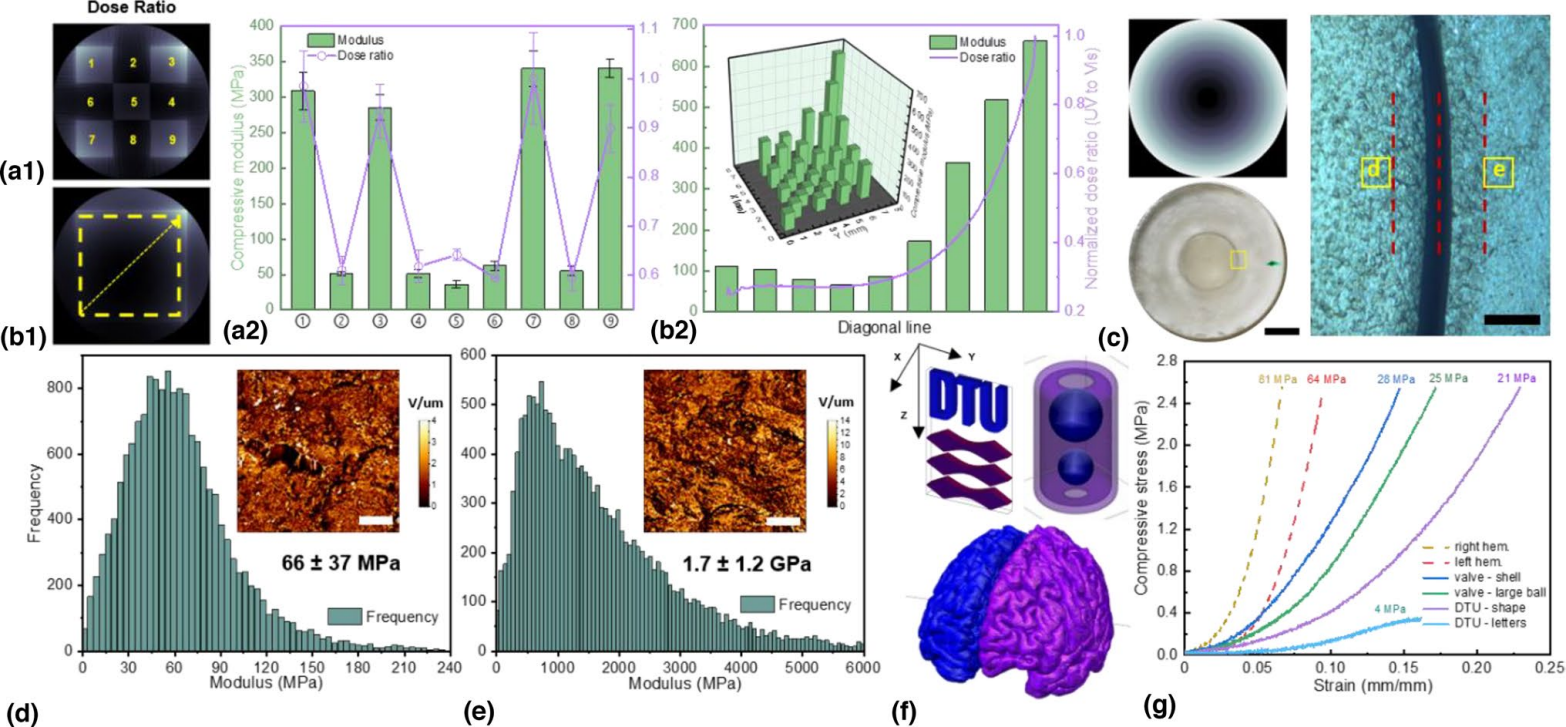
Property modulation via orthogonal photo-polymerization. (a) Stiffness of end-product correlates strongly with UV-to-visible dose ratio. (b) A continuous property gradient can be generated laterally by using greyscale printing to modulate the relative extent of conversion for free radical- and cationic polymerization. (c) The radial resolution of stiffness control is 300 µm or better, comparable to the printing resolution of the same TVAM setup (scale bars = left 3 mm, right 150 μm). (d) and (e) Analysis using atomic force microscope reveals an achievable modulus gradient of 5 MPa/µm. (f) and (g) Dual color property modulation can be realized in structures challenging to print using conventional AM methods. (Copyright pringer-Nature).^[^^17^^]^

Stiffness modulation by multiwavelength TVAM is viable thanks to photolysis of cationic PI also generating free radicals and polymerization of epoxide being slower than that of acrylate. Slow superacid propagation avoids immediate over-exposure of out-of-part voxels. UV triggers free radical polymerization, meaning that the principle underpinning geometric shaping, which relies on contrast-building via pre-existing free radical quenchers, remains valid regardless of the incident wavelength. Wang et al*.*^[17]^ combined the formulation of Larsen et al*.*^[64]^ and Kelly et al*.*^[9]^ into a wavelength-sensitive photoresins and used a two-color VAM setup (*λ*_1_ = 455 nm, *λ*_2_ = 365 nm) to generate internal mechanical property gradients. It was first established that a strong correlation exists between the UV-to-visible dose ratio and the stiffness of the end product [Fig. 3(a)]. This correlation may be combined with greyscale printing to generate a continuous property gradient in the lateral plane [Fig. 3(b)]. In the radial direction, stepwise stiffness changes were created within 300 µm, comparable to the printing resolution of the setup used [Fig. 3(c)]. A detailed surface analysis in selected regions of interest [Fig. 3(d) and (e)] revealed that an average gradient of 5 MPa µm^−1^ could be achieved. The geometric design freedom of VAM was exploited to fabricate objects with suspending and enclosed structures without support struts [Fig. 3(f)], and with stiffness modulation in their intricate geometries [Fig. 3(g)]. Generating the patterns for a balanced print fidelity and property contrast via dual-color greyscale printing proved challenging.

### Sintered materials

Ceramics and glasses are materials with remarkable properties like hardness, thermal resistance, chemical resistance and inertness, and, in the case of glass, optical transparency and refractivity. Thanks to these properties, ceramics and glasses have numerous industrial and technical applications, from everyday kitchenware to implantable prosthetics and insulators for outterspace satellites. It is also because of their high mechanical resistance and brittleness, that these materials are difficult to shape into complex geometries, particulary by means of traditional subtractive manufacturing.^[69,70]^ Recently, developments in additive manufacturing have opened new possibilities for the fabrication of objects and devices with greater design freedom from glass and ceramics.

The fabrication of ceramics and glasses requires high temperatures or pressures to give the desired mechanical, chemical, or thermal properties to the material. Plastics and organics are, on the other hand, easy to shape; although they lack mechanical, chemical, and thermal resistance.

**Figure 4:**
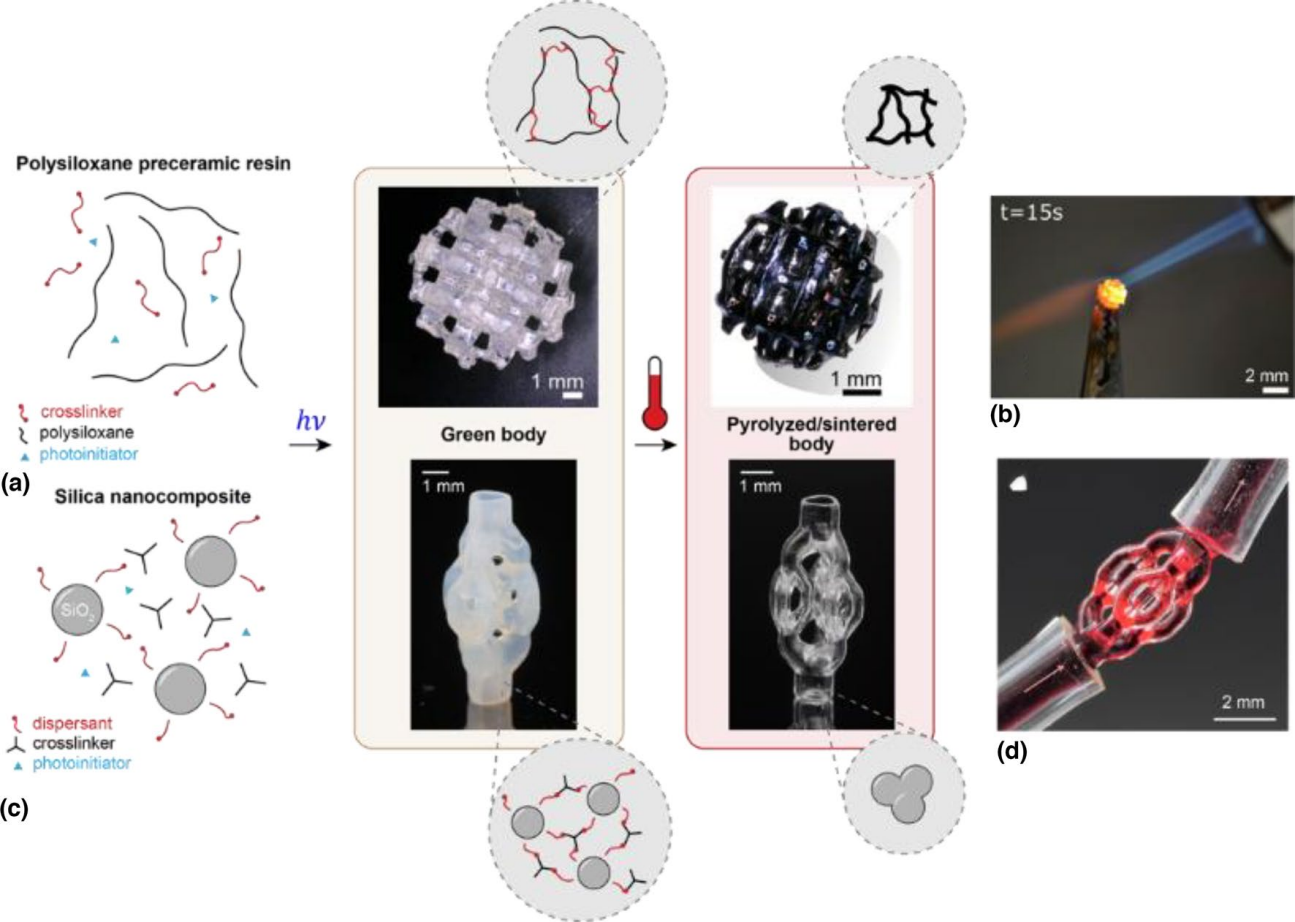
Volumetric additive manufacturing of ceramics and glasses. (a) To fabricate ceramics, a photo-curable polysiloxane preceramic resin is shaped by exposing it to light in a volumetric printer. The resulting the printed green body, has a cross-linked organic mesh at its microscale. Then, the green body is pyrolsyed at 1000°C. At these high temperatures, the organic moieties of the preceramic resin are burnt and evaporated away from the green body, leaving an amorphous inorganic SiOC ceramic part. (b) The transformation makes the object highly temperature resistant.^[16]^ (c) A nanocomposite resin composed of silica nanoparticles coated with a dispersant, an acrylic crosslinker and a photoinitiator is shaped with light in the volumetric printer. After sintering the green body at 1300°C, (d) transparent glass devices, like this microfluidic structure, are obtained.^[11]^

In the additive manufacturing of ceramics and glasses, a well-established strategy is to combine the shapeability of plastics and the resistance of ceramics by fabricating a softer version of the object, also know as the green body. The green body is rich in organics and has low stiffness, but has already been molded to the desired shape. The green body can be formed from polymer derived ceramics or nanocomposite suspensions, as we will cover in this section. Then, it can be pyrolyzed or sintered at high temperatures in a furnace, usually above 800°C, in a process that burns all the organic components and leaves the inorganic ceramic or glass behind, as shown in Fig. 4. Pyrolisis or sintering comes with shrinkage and mass loss; thus reducing cracks from burning the organic part is an active field of study.^[71]^

#### Polymer derived ceramics

The fabrication of polymer-derived silicon-based objects by the pyrolsis of organosilicon polymers was developped in the 1960s.^[69]^ Since then, numerous ways to pyrolyze these polymers into ceramic parts have been presented and the library of materials has grown from binary systems as SiC and SiN to more complex systems like SiCN, SiCO, SiBCN, SiBCO, and SiAlCO. Pyrolyzing polymeric precursors requires typically lower temperatures (900–1100°C) than sintering ceramics from powders (1700–2000°C).

During pyrolysis, organic moieties are eliminated by breaking the lower-energy C-H bonds; releasing gases and leading to mass loss and shrinkage.^[69]^ However, when gases cannot escape rapidly enough from the bulk of the greenbody, pressure increases rapidly thanks to high temperature, which results in the formation of cracks. Cracks are generally undesirable and are responsible for low yield rates of sintering processes. To minimize the formation of cracks, sintered objects are designed to be thin (< 1 mm) or porous,^[72]^ and they are sintered following long, slowly-increasing heating profiles with holding steps at the temperatures of solvent evaporation and polymer decomposition.

Although polymeric precursors can be formed by molding or thermally-induced gelling; the advent of photocurable materials has enabled the shaping of ceramics with higher precision and flexibility. Introduced by Liew et al*.*, the UV photopolymerization of a polysilazane allowed the fabrication of SiCN microelectromechanical systems using lithographic masks.^[73]^ More recently, Zanchetta et al*.* demonstrated the stereolithographic fabrication of crack-free SiOC microcomponents from polysiloxanes;^[74]^ which are insensitive to air and moisture, unlike polysilazanes. The method has been expanded to fabricate multi-cm heat shields using high-area rapid printing (HARP)^[3]^ or crack-free micrometric injectors using two-photon polymerization.^[75]^

Kollep et al*.* demonstrated that VAM could be also applicable to polymer-derived ceramics.^[16]^ They developed an optically clear resin composed of a commercial polysiloxane and 1,4-butanediol diacrylate as a crosslinker with diphenyl(2,4,6-trimethylbenzoyl)phosphine oxide (TPO) as photoinitiator. The crosslinker reduced printing times while photoinitiator concentration could be kept low. The resulting printed green bodies exhibited resolutions down to 80 μm. They were then pyrolysed at 1000°C, yielding dense ceramic componets, with smooth surfaces and an isotropic linear shrinkage of 31% and a mass loss of 54%. Additional Raman, FTIR, and X-Ray Photoelectron Spectroscopy demonstrated the conversion from an organic green body to an inorganic SiOC ceramic with intermediate mixed silicon oxycarbide species and free-carbon intrusions.^[76,77]^ The fabricated components, including screws and spherical woodpiles, exhibited high thermal and chemical resistance after being subject to heating cycles up to 1400°C and being immersed in corrosive acidic (pH 2) and basic (pH 14) baths.

#### Glass

Traditionally, glass has been shaped by forming technologies like blowing and casting or subtractive methods like grinding and chemical etching. Freeform 3D shape control of glass originated with molten glass filament deposition^[78,79]^ and powder-based laser sintering.^[80]^ These, being direct structuring processes, require localized high temperatures to sinter or melt the feedstock at the time of printing. Alternatively, multistep processes transform a body of pre-glass polymer^[81–84]^ or hybrid glass–polymer nanocomposite^[85–87]^–shaped by low-temperature 3D printing methods–into high-purity glass with a thermal treatment. These materials are often designed to be compatible with existing 3D printing technologies like direct ink writing (DIW), stereolithography, and digital light printing (DLP). Fidelity can be improved over high-temperature direct structuring; however, for layer-by-layer processing the rheological properties of the liquid precursor material must be optimized. For instance, desirable properties include shear-thinning for recoating in DLP and fast viscoelastic recovery to retain shape after extrusion in DIW. Moreover, complex geometries may require support structures which can limit geometric freedom and lead to surface artifacts and roughness which may, at best, require further polishing and, at worst, create anisotropic mechanical properties.

Volumetric printing has important advantages for glass printing. Lack of fluid motion permits high solids loading, high-viscosity precursor liquids, and layer-less volume-at-once formation. Furthermore, high particle loading allows components of medium molecular weight to sharply transition from a sol to solid object thus requiring less dose and thus lower irradiation times than bulk polymerization without solid loading. As sufficient material transparency at the digital projection wavelength is a necessary condition for tomographic VAM, the two phases of a particle-loaded precursor―solid silica nanoparticles and liquid monomer binder―should be nearly refractive index-matched. This strategy was used in the first demonstration of transparent glass tomographic VAM printing;^[11]^ a nanocomposite formulated for DLP^[85]^ was adapted to volumetric printing by including a radical quencher (see section *Thiol-enes*) to improve polymer conversion contrast for microscale printing. Complex structures were fabricated including periodic lattices and 3D branched microfluidic pathways demonstrating minimum positive and negative feature sizes of 50 µm and 150 µm, respectively. Multielement lens systems and microlens arrays exhibited minimum R_a_ roughness of 6 nm without additional polishing steps, and fair optical performance. Preliminary comparison of flexural strength of solid beams printed volumetrically to DLP-printed beams showed enhanced Weibull modulus, i.e. decreased variation in modulus of rupture, and smaller average and standard deviation peak-to-valley roughness, both suggesting more uniform and smaller defects were present in beams produced by VAM.

Early results show the potential of volumetric printing for glass components, but sub-millimeter maximum cross-sectional thickness and single-material silica composition could impede adoption for certain important applications. Crucial to formulation of a suitable resin that enables centimeter-scale cross-sections are proper dispersion of silica into resin, index matching of silica to resin, tuning of solvents that enable drying without cracking, and development of a suitable polymer binder system. Prior resin formulations used in VAM and DLP achieve this through the use of monofunctional monomers, such as hydroxyethyl methacrylate (HEMA), that serve as a polymer binder while also enabling proper mixing of silica through the formation of a solvation layer.^[85,88]^ However, the non-uniform polymerization of monofunctional monomers in centimeter-scale parts results in a wide distribution of the molecular weight products. Low molecular weight products can greatly disrupt the drying process. Polymerization of multifunctional monomers and oligomers mitigates this effect, thus enabling larger parts with more uniform drying, though monomer choice must be optimized to avoid shrinkage-induced stress and warping. Beyond single-material silica composition, advanced glass applications like scratch or impact resistant glass, gradient refractive index lenses, and optical fibers require different oxide compositions or even gradients of multiple oxides including Al_2_O_3_, B_2_O_3_, AlNaO_6_Si_2_, TiO_2_, and PbO.^[89]^ Although matching the refractive index of SiO_2_ is easily accomplished with readily available monomers, index-matching high refractive index glasses, like metal oxide and chalcogenide glasses (*n*_D_ > 2), will be challenging. Alternatively, doping with TiO_2_, GeO_2_, and Ag nanoparticles can enable tuning of glasses with refractive indices up to 1.58.^[83,84,90]^ High-index sulfur-containing monomers^[91]^ could be a candidate for nanocomposites containing glasses up to n_D_ ≈ 1.7–1.8. Red-shifting the actinic wavelength into infrared until a match can be found or even into microwave where scattering will be significantly reduced^[55]^ may be required for still higher refractive index glass. Light propagation models which account for disordered media offer an alternative strategy when mitigation of light scattering is not possible. Software-based light scattering compensation of amplitude projections has resulted in improved VAM print fidelity for low concentrations of TiO_2_^[34]^ and wavefront shaping, which has proved indispensable for deep photostimulation in scattering biological tissues,^[92]^ could be transferred to VAM for phase-modulated volumetric printing.

Inorganic alkoxide precursors^[93]^ which allow deliberate control of composition and creation of glass and ceramic by the sol–gel process^[94]^ represent a second class of transformable materials compatible with photopolymerization-based 3D printing which may circumvent the refractive index matching challenges of the nanocomposite approach. In the context of tomographic VAM, using preceramic precursors to print silicon oxycarbide ceramics has already been reported (see section Polymer derived ceramics);^[16]^ however, the field of sol–gel processing is rich for exploration, especially for volumetric printing of multicomponent glass.^[95]^ Moore et al*.* introduced a resin for DLP 3D printing composed of silicon, phosphorous, and boron alkoxide precursors and organic monomers that achieved photopolymerization-induced phase separation and intensity-controllable pore size.^[96]^ This type of greyscale control over properties will be important for advanced spatial control of composition in tomographic VAM that comes naturally with other 3D printing processes like DIW.^[83]^ More generally, by inducing the condensation reaction via elevated temperature *after* the printing process, formation of solids can be delayed until after printing. This approach could be beneficial for volumetric printing of high index glasses as the refractive index matching requirement would be obviated. In addition to bulk glasses, omitting sintering to full density, the intermediate porous aero or xerogel products of the dehydration process can be investigated for applications like filtration, gas capture, and insulation.

### Thiol-enes

In 2001, Sharpless et al*.* introduced the concept of click chemistry defining modular, orthogonal highly efficient reactions with non-toxic end products and mild running conditions.^[97]^ When it comes to photochemical click reactions, or simply photoclick reactions,^[98,99]^ the step-growth thiol-ene mechanism represents the most widely used.^[^^100^^,101–103]^ The family of possible polymers is vast because a large library of thiols can be paired with a similarly large family of electron-rich or strained enes. The regioselective propagation reaction follows a step growth process, delaying gelation to dramatically reduce curing stress and shrinkage in comparison to chain growth processes common to acrylates.^[104–106]^ Polymerization rates are strongly controlled by the ratio of kinetic constants between the thiyl radical propagation and the chain transfer from a carbon-centered radical to a thiol.^[107]^ Thiol-ene networks are significantly more uniform than those formed by acrylates,^[106,108]^ exhibiting much narrower glass transitions temperature ranges, forming homogeneous gels, elastomers and glasses.^[109]^ These materials generally have high optical clarity and refractive index, making them particularly suited to optical applications.

Thiol-ene polymers also readily support additional processes after photopolymerization that can modify their properties. Since the step-growth reaction proceeds by alternation to nearly full conversion with no homopolymerization, addition of a stoichiometric excess of thiols or enes results in remaining reactive groups for later reaction stages.^[110]^ The thiol-thioester exchange reaction or the disulfide addition–fragmentation chain transfer reaction can locally transform the elastic thermoset network to exhibit viscoelastic plastic flow.^[111]^ Finally, thiol-ene polymers can be engineered as high performance shape memory materials with excellent shape fixity and rapid actuation.^[112]^

Thiol-enes have several specific advantages when used in volumetric additive manufacturing and one disadvantage. Advantageously, the rapid reaction times of thiol-enes, particularly when using strained enes such as norbornene, can be as fast as 1–10 s, matching the potential rate of VAM printing. Additionally resins and hydrogels made through these fast photoclick chemistries may potentially have higher biocompatibility compared to acrylate-based systems, improving cell viability and biodegradability in constructs fabricated through volumetric bioprinting (see section Hydrogels & cell-laden hydrogels).^[15,19,22]^ VAM resins are also completely contained in a closed volume, alleviating the odors that arise from low molecular weight thiol monomers. Conversely, the insensitivity of thiol-ene reactions to oxygen makes VAM printing more challenging, since this inhibitory threshold is used in acrylate systems to suppress gelation outside of the desired print volume. Additionally, similarly to the large refractive index changes seen in (meth)acrylate chain-growth polymerization reactions, refractive index changes during thiol-enes polymerization can result in self-focusing waveguide formation during VAM,^[43]^ impacting print quality.

**Figure 5:**
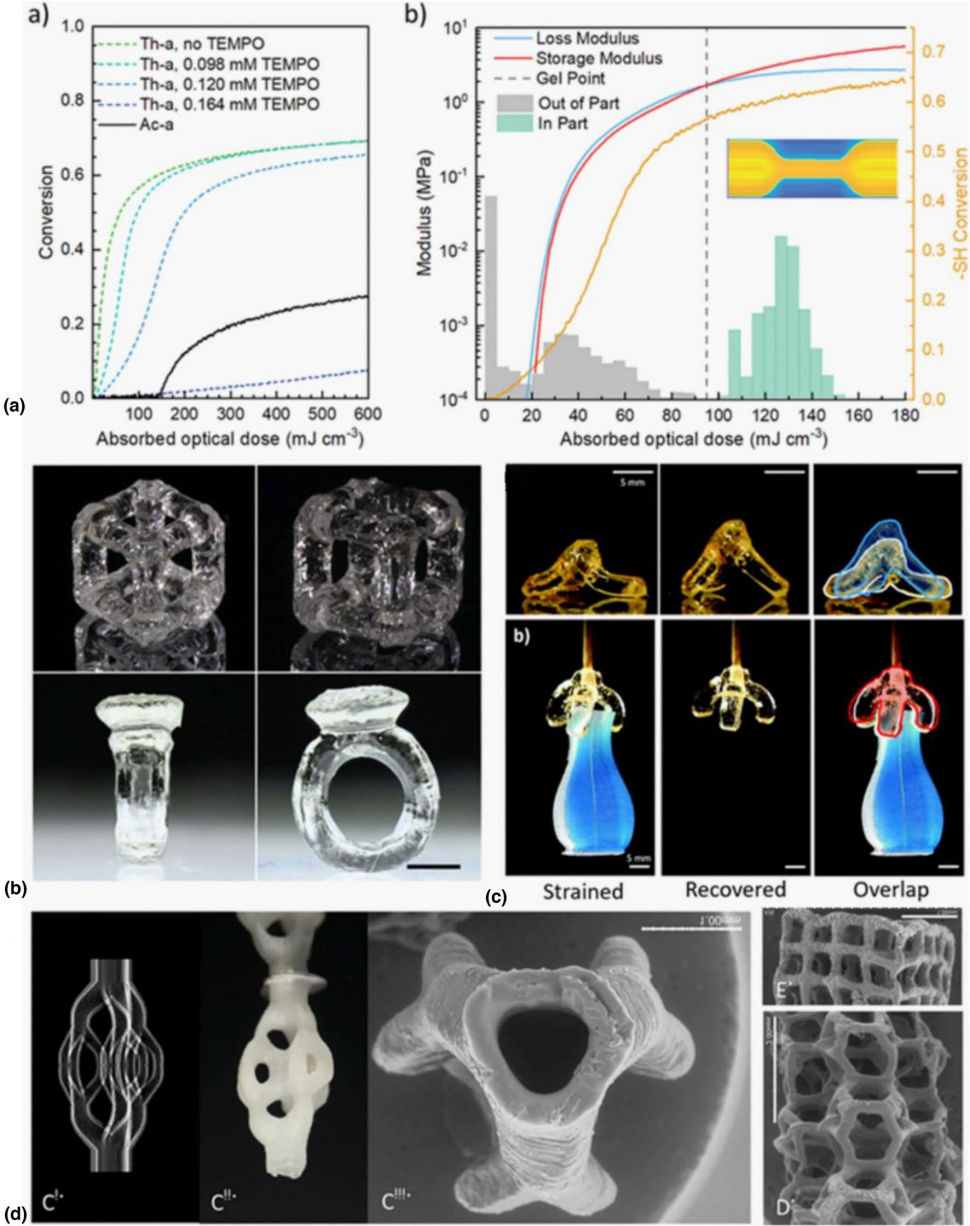
Volumetric Additive Manufacturing of thiol-ene photoresins. (Aa) Conversion response to light dose in thiol-enes. Unlike acrylates, thiol-enes are not subject to polymerization inhibition from molecular oxygen; instead, by adding TEMPO, a radical scavenger, conversion can be thresholded. (Ab) Mechanical properties (storage and loss moduli) of thiol-enes are tunable along a large dynamic range. (b) Resulting prints, exhibiting overhangs and cavities (scale bar = 5 mm, Copyright Wiley).^[13]^ Thiol-ene printed parts can be thermally treated to exploit shape memory effects, as in this tripod and gripper (scale bars = 5 mm, Copyright Wiley).^[14]^ (d, ci) One of the light patterns used to generate (cii) poly(ε-caprolactone) microfluidic devices and (d) and (e) geometric lattices (scale bars = 1 mm, 2 mm & 1 mm, respectively; Copyright Wiley).^[15]^

In order to induce the necessary thresholding within thiol-ene polymerizations for VAM, small concentrations (~ 0.1 mM) of 2,2,6,6-tetramethyl-1-piperidinyloxy (TEMPO) as a radical scavenger were found to be sufficient.^[13]^ TEMPO, however, also retards the overall cure speed and delays gelation, so these competing effects must be carefully balanced. In addition, the threshold provided by TEMPO is “softer” than that seen with O_2_ in acrylate systems [Fig. 5(a)], requiring careful tuning of photo-dosing relative to reaction kinetics. Another challenge in adapting thiol-ene polymerizations for VAM is their propensity to thermally cure under ambient conditions, reducing vat pot-life. Additions of pot-life stabilizers such as ANPHA were found to increase stability of the resin at elevated temperatures and room temperature and improve handling for printing.^[14]^ With these considerations in mind, thiol-ene resins for VAM were formulated with trifunctional thiol monomers and varying rigid (a trifunctional isocyanurate ring (ICN)) and flexible (a triethylene glycol chain (TEG)) ene monomers for tunable mechanical response.^[13,14]^ Photorheological and real-time photo-FTIR analysis was used to identify the critical volumetric energy dose and gelation threshold for printed resins for generation of the tomographic projections. Unlike comparable acrylate compositions, the mechanical responses of thiol-ene systems are highly tunable in relation to the ratio of rigid and flexible ene monomers, with elastic moduli spanning three orders of magnitude (421 to 0.12 MPa), and ultimate strains ranging from 44 to 450% depending on the formulation. VAM printing of these thiol-ene resins confirmed the expected highly tunable and robust mechanical properties compared to chain-growth acrylate counterparts.^[13]^ Additionally, thiol-ene materials have improved biocompatibility and unique shape memory behavior, providing enticing avenues to target soft robotic and biological applications.^[13–15]^ Ultimately, the uniformity of the thiol-ene polymer networks opens up a massive modifiable material toolkit for VAM, as simple changes to monomer and choice of thiol and ene functionality directly impact the bulk material and mechanical properties.^[15]^

Among the unique material properties identified in thiol-ene VAM materials, shape memory behavior is appealing for developing responsive or active materials. Shape memory polymers (SMP) can be physically deformed to a “temporary” shape, then recover a more “permanent” state in response to external stimuli such as temperature, pH, light and electromagnetic fields. The most easily programmed and well-studied of these is temperature, as shape memory behavior is easily controlled by heating the shape memory polymers above its glass transition temperature (*T*_g_).

Schwartz et al*.* adapted thiol-ene formulations to fabricate devices with facile, controlled thermal programming of shape memory polymers behavior.^[14]^ The trifunctional thiol component was kept constant in their photoresins; yet they varied the amount of flexible and rigid bifunctional and trifunctional ene monomer-subunits to modulate ultimate tensile strain and *T*_g_. Dynamic mechanical analysis showed that the components exhibited nearly full shape recovery over four repetitions at strains up to 18.4%, similar to skeletal muscle. Using these characterized thiol-ene materials, the authors printed a three-arm gripper and self-standing tripod structures by VAM (Fig. 5). After printing, structures were programmed to their temporary shape through heating above the *T*_g_ to 80°C and deforming the structure before cooling. Upon heating above the *T*_g_ again, the gripper and tripod structures recovered to their permanent printed configuration. As VAM produces 3D structures without layering, the shape memory behavior was isotropic, unlike traditional layer-based AM methods where print orientation during printing can imbue anisotropies. [113].

Photoclick thiol-ene chemistry has also been applied to volumetrically print poly(*ε*-caprolactone) (PCL) constructs with improved mechanical properties and lower brittleness than most acrylate cross-linked counterparts.^[15]^ PCL is degradable and biocompatible, making it the material of choice for many resorbable synthetic implants in the past decades. Thijssen et al*.* have shown that volumetrically printed thiol-ene PCL constructs, including lattices and branching tubular structures (Fig. 5), were non-toxic and biocompatible in vitro and in vivo. Volumetrically printed PCL could be a promising candidate for the generation of complex cell-free 3D implants.

### Hydrogels and cell-laden hydrogels

The past two decades saw the emergence of the field of biofabrication,^[114,115]^ in which additive manufacturing techniques have been engineered to process also living cells and cell-laden materials. Typically, these materials are in the form of hydrogels, aqueous networks of hydrophilic polymers that allow for the embedding of cells and preserve their viability and functionality, acting also as temporary analogues of the native extracellular matrix (ECM) present in living tissues. In the context of biofabrication, cell-laden hydrogels are termed bioinks or bioresins (when used for extrusion or light based printing techniques, respectively).^[116,117]^ If cells are added to the hydrogel only after printing, the term biomaterial inks is instead commonly used.^[116]^ The unique ability of biofabrication techniques to control the spatial positioning of cells, bioactive molecules and biomaterials, is being leveraged by biomedical scientists to engineer tissues that mimic the anatomical composition and salient functions of native organs and biological systems.^[118]^ Key applications include the production of living grafts for regenerative medicine,^[119]^ the generation of tissue models as alternatives to animal experimentation for pharmaceutical and biological research,^[120]^ as well as engineered living materials as biotechnological products.^[121]^

In this context, the bioprinting process needs to be carefully designed not to harm cell integrity, viability, and health during and after printing. Extended printing times (from tens of minutes to hours), necessary to fabricate centimeter-scale, clinically-relevant structures are characteristic of conventional layer-by-layer bioprinting methods; which may impair cell functionality if cells are kept outside of their optimal culture environment for too long.^[122]^ Addressing this drawback of classic additive manufacturing approaches, Bernal et al*.* first introduced the concept of Volumetric Bioprinting (VBP), generating cell-laden, functional tissues in few seconds, by means of tomographic printing.^[10]^ VBP is simply VAM applied to fabricate cell-laden constructs for tissue engineering and tissue modelling. As photo-responsive bioresin component, gelatin methacryloyl (GelMA) was used for this first study, and since then, the library of materials available for volumetric bioprinting has rapidly expanded. The following section will review the main materials and chemistries applied in and developed for this printing technology.

#### GelMA

Gelatin, a biopolymer obtained from collagen denaturation, has been widely used as biomaterial for tissue engineering and bioprinting. This is due to the material’s biocompatibility, controllable degradation profile, and its ability to promote cell adhesion and several cellular functions.^[10]^ While gelatin undergoes physical gelation when cooled below body temperature, at 37°C the polymer is fully soluble in aqueous solutions. Therefore, thermostable hydrogels with several crosslinking modalities can be obtained, for instance through the introduction of photo-responsive moieties.

The first report on the synthesis and utilization of (meth)acryloyl modified gelatin, named GelMA, appeared in the year 2000.^[123]^ In the presence of a photoinitator, the (meth)acryloyl moieties undergo free-radical polymerization, rapidly forming a covalently crosslinked hydrogel, whose mechanical profile can be tuned by varying the GelMA pre-polymer content and the degree of methacryloyl substitution.^[124]^ Most notably, GelMA is readily accessible to many research labs through commercially available products or in-house synthesis, following well-described protocols.^[124,125]^ Moreover, medical-grade, endotoxin-free material processing routes have been established, therefore facilitating the potential translation of GelMA-based constructs towards pharmaceutical and medical products.^[126]^ As such, GelMA rapidly became one of the most widely used bioinks for extrusion printing,^[127–129]^ and more recently also for light-based bioprinting.^[117,130]^

**Figure 6:**
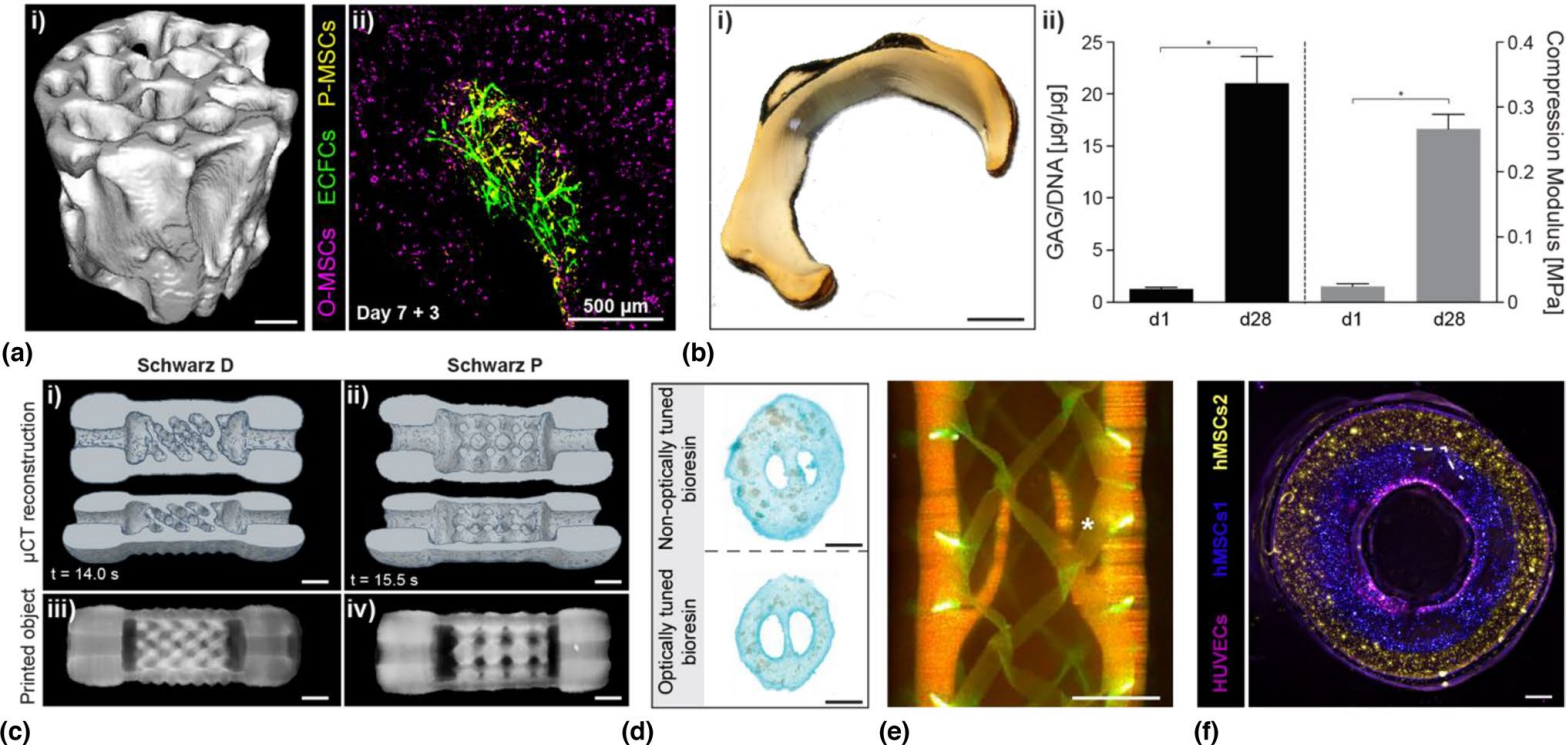
Hydrogels & cell-laden hydrogels: (ai) 3D micro-CT reconstruction of a native trabecular bone model printed via VBP with a 10% GelMA bioresin (scale bar = 2 mm). (aii) MSC/ECFC co-culture seeded into the pores of the MSC-laden trabecular bone model showing early endothelial cell infiltration into the printed hydrogel (scale bar = 500 μm). (b) Digital model of a VBP-printed meniscus model (10% GelMA bioresin) cultured for 28 days and (ii) exhibiting significant increases in neocartilage matrix production and compressive properties. Adapted from Ref. 10. (c) Organoid-laden VBP of convoluted, mathematically derived Schwarz D and P structures as shown in (i) 3D micro-CT reconstructions and (ii) digital images (scale bars = 2 mm). (d) Stereomicroscopy images of non-optically tuned 5% GelMA and iodixanol-supplemented bioresins for organoid VBP (scale bars = 2 mm). Adapted from Ref. 21. (e) VBP of GelMA bioresins into a venous valve structure (red) throughout an opaque melt electrowritten tubular mesh (green) (scale bar = 2 mm). (f) Multi-cellular, multi-layered tubular construct reinforced with tubular melt electrowritten meshes (dotted line) (Scale bar = 500 μm). Adapted from Ref. 23.

In 2019, Bernal et al*.*, developed a GelMA-based bioresin for tomographic volumetric bioprinting, using lithium phenyl-2,4,6-trimethylbenzoyl-phosphinate (LAP) as visible-light type I initiator (0.037% wt in PBS).^[10]^ The system facilitated the generation of anatomical, centimeter-sized trabecular bone models embedding mesenchymal stromal cells (MSC) in 12.5 s, with high cell viability (> 85%), comparable to non-printed controls [Fig. 6(Ai)] The bioprinted cells remained functional and ingrowth of capillary-like structures from seeded endothelial cells and pericytes, was observed in the printed hydrogel [Fig. 6(Aii)]. By converting GelMA into a thermoreversible gel form prior to printing, cells remained homogenously suspended in the bioresin, and no artefacts due to sedimentation were experienced, with ~ 95% volumetric fidelity compared to the original STL files. Moreover, further underlining the feasibility and potential of VBP, long-term (28 days) culture of articular cartilage progenitor cells (ACPCs) was shown, by bioprinting 10 million cells mL^−1^ into a meniscus-shaped construct Fig. 6(Bi)]. Exposed to chondrogenic media, ACPCs readily colonized the hydrogel with fibrocartilage-like matrix, which endowed the constructs with compressive stiffnesses comparable to that of human knee menisci [~ 0.3 MPa; Fig. 6(Bii)].^[10]^ Long-term culture was also later shown by Gehlen et al*.*, in the context of bone tissue engineering, demonstrating the expression of osteoblastic and osteocytic markers in bioprinted MSC-laden GelMA over 42 days.^[21]^

Besides offering a platform to engineer connective tissues, low stiffness (compressive modulus < 2 kPa) GelMA bioresins for VBP have also been optimized to culture organoids, which are miniaturized, multicellular 3D structures in which cells self-organize to capture organ-like behavior. The nozzle-free, shear stress-free nature of VBP allowed to process the organoids, which otherwise tend to fragment during extrusion-based bioprinting,^[131]^ into complex perfusable hepatic constructs capable to express key functions of the native liver, as shown by the ability to detoxify ammonia over the course of a dynamic flow experiment [Fig. 6(c)]. The geometry imposed by VBP could be varied to tune the rate of ammonia conversion to urea, underlining how bioprinting and its freedom of design can be leveraged to tune the functionality of engineered tissues.^[20]^ Notably, in the same study, Bernal et al*.* demonstrated that supplementing the bioresin formulation with the biocompatible compound iodixanol,^[132]^ the refractive index of GelMA could be tuned to match that of intracellular organelles, therefore minimizing the negative effects that cell-mediated light scattering can cause on printing quality when using high cell densities. Defect-free prints with 40 µm-features, the highest resolution achieved in the presence of cells to date, were thus shown for constructs laden with up to 1.5 · 10^7^ cells mL^−1^.^[20]^ While further increasing the cell content will likely require additional corrective strategies, iodixanol supplementation is a simple and versatile tool applicable for all types of bioresins [Fig. 6(d)]. In another soft tissue application, Sgarminato et al*.* fabricated fibroblast-laden duct-like structures in GelMA, where they seeded human pancreatic epithelial cells inside the duct and studied the inflammatory response of these cells in a tumor microenvironment.^[45]^

The thermoreversible behavior of GelMA prior to covalent (photo)crosslinking is also beneficial to print composite (bio)materials, in which pre-formed objects are embedded within the bioresin, and secured in place once the thermal gel is formed. Groessbacher et al*.*, utilized this concept to demonstrate the possibility of sculpting (cell-laden) GelMA in presence of opaque, poly(*ε*-caprolactone) fiber meshes produced by melt electrowriting.^[23]^ Multi-material and multi-cellular prints were produced mimicking the architecture of blood vessels, and structures with varying degree of architectural complexity (venous valves, branched structures, partly occluded vessels) were obtained via volumetric printing [Fig. 6(e), (f)]. The combination of GelMA as a bioprintable matrix for cell culture with stiffer thermoplastic polymermeshes also constitutes a promising strategy for modulating the mechanical properties of the composite construct, as shown with vessel-like prints approximating the mechanical performance of porcine coronary arteries.^[23]^ Further optimization of volumetrically-printed GelMA constructs can also follow the selection of the source gelatin material (mammals, fish, or recombinant), exploring different degrees of methacrylation to facilitate in-gel cell migration and vascular formation,^[133]^ and the further functionalization with other (light-responsive) chemical groups. In this line, Soliman et al*.*, recently reported the multi-material volumetric printing of GelMA perfusable gyroids, within which plugs of pristine, unmodified gelatin were printed utilizing tris(2,2′-bipyridyl)ruthenium (II)/sodium persulfate (Ru/SPS) as initiator.^[134,135]^ This ruthenium complex, a type II initiator, can trigger di-tyrosine oxidation, therefore catalyzing the formation of covalent bonds between aromatic side chains in gelatin. While this photochemistry could be used in combination with GelMA, exploiting either the native tyrosine residues, or adding tyramine-like groups onto the polymer, [136] it could also be used for the volumetric printing of tyraminated polymers, or even unmodified proteins and peptides.

#### GelNB and photoclick biopolymers

As introduced in Sect. "Thiol-enes", in the context of photoclick reactions, the step-growth thiol-ene mechanism represents the most widely used in this field. Among the possible -ene groups, the use of norbornene (NB) offers significant advantages for tissue engineering and bioprinting.^[99,137]^ In particular, the NB functionality has the advantage of being insusceptible to Michael-type addition (unlike the more traditional acryloyl and methacryloyl moieties); while its ring-strain conformation makes it highly reactive upon formation of a thiyl radical. Overall, the use of the photoclick thiol-ene step-growth crosslinking mechanism comes with a series of biologically relevant advantages when compared to chain-growth systems (i.e., (meth)acryloyl derivatives such a GelMA and PEGDA).^[104,109,138]^ Due to higher reactivity and insensitivity to oxygen, thiol-ene networks are formed faster, thus reducing the light exposure time and the potentially cytotoxic radical initiating species.^[139]^ In contrast, chain-growth polymerization occurs by creating hydrophobic kinetic chains that are non-biodegradable. This process leads to the formation of heterogeneous networks and volume shrinkage, which are undesirable phenomena.

**Figure 7:**
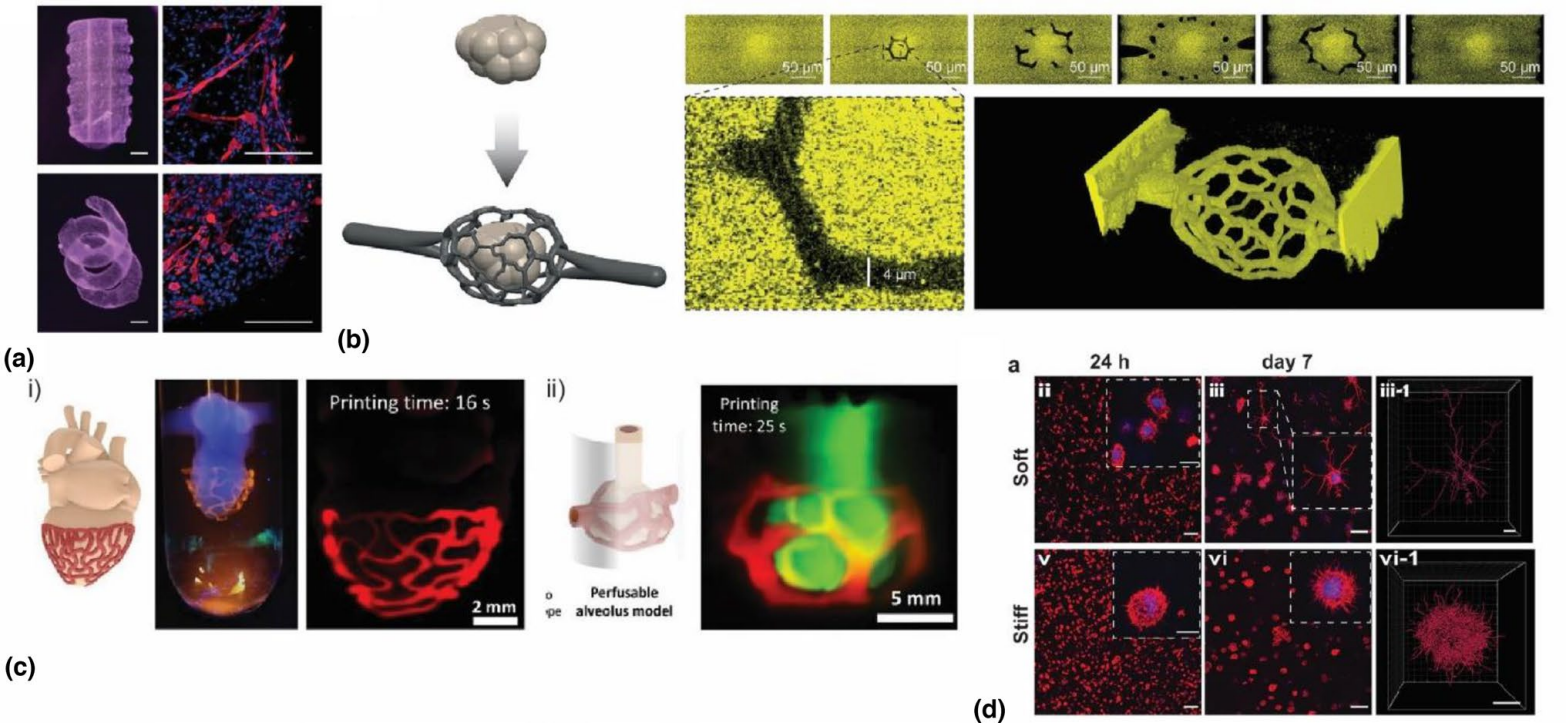
VAM of GelNB hydrogels**. **(a) VBP of complex geometries in the presence of myoblasts showing spreading and differentiation into myotubes (Myosin Heavy Chain: red, Nuclei: blue) Scale bars: left 2 mm, close-ups right 200 µm^[19]^ (b) Two-photon ablated microchannels connecting cavities printed by VAM (Copyright Rizzo et al*.*)**.**^[144]^ (c) Algorithmically designed organ-specific auxetic meshes and perfusable networks volumetrically printed around pre-existing constructs. (Copyright Chansoria et al.).^[145]^ (d) Cell spreading in VBP printed PVA-NB hydrogels of varying stiffness (scale bars: 100 µm and close-ups 50 µm copyright Wiley).^[22]^

In the context of VBP, thiol-NB chemistry has been first introduced by Rizzo et al*.* with a photoresin composed of gelatin norbornene (GelNB) and 4-arm-PEG-thiol (PEG4SH).^[19]^ As previously discussed, gelatin possesses both mechanical and biological desirable properties for VBP and thus represents an ideal material platform for photoclick crosslinking. Thanks to the faster crosslinking of photoclick chemistry, printing times for centimeter-size objects in this GelNB/PEG4SH bioresin were reduced to ~ 10 s [Fig. 7(a)]. Lower light exposures and generation of radical species resulted in excellent cell viability of embedded cells (> 95%). In addition, thiol-NB crosslinking made it possible to combine such printing performances with a lower gelatin degree of substitution and concentration, allowing to retain the native polymer properties,^[123,140]^ and to generate softer matrices known to result in enhanced nutrient diffusion and cell migration capabilities.^[141–143]^

Although thiolated PEG is a commonly used bioinert thiol-ene crosslinker, the literature provides methods for synthesizing and functionalizing thiol- polymers of various types, including synthetic and naturally-derived polymers. This presents an opportunity to customize the thiol-ene photoresin properties further, biophysically and biochemically. For example, GelNB has been recently combined with thiolated gelatin (GelSH) to obtain a purely gelatin-based VBP photoresin.^[144,145]^ In a first work, VBP printing of perfusable constructs was reported with an overall polymer concentration of only 2.5% w/v.^[144]^ Interestingly, the use of a gelatin-only photoresin made the resulting hydrogel well susceptible to two-photon ablation (2PA); converging VBP and 2PA for the first time. Using this hybrid method, the authors overcame the resolution limit of VBP and created multiscale complex perfusable geometries with features spanning from hundreds (VBP) to few microns (2PA) [Fig. 7(b)]. Leveraging VBP's unique capability to 3D print around a preexisting shape,^[9,23,145]^ GelNB/GelSH photoclick bioresin was also employed by Chansoria et al*.* to produce organ-specific auxetic meshes wrapped around a heart as well as perfusable geometries enveloping an alveolus model [Fig. 7(c)].^[145]^

Although gelatin has so far prevailed as the preferred material choice for VBP, a recent work from Qin et al*.* has expanded the library of VBP bioresins by introducing NB polyvinyl alcohol (PVA) derivatives.^[22]^ The authors used chemically unmodified gelatin to guarantee reversible thermal gelation, thus eliminating sedimentation-induced artifacts, and at the same time as sacrificial material to leave behind a soft, cell permissive PVA-based network upon thermal removal. In this regard, VBP is foreseen to play a central role in tackling the challenge of biofabricating soft tissues with structural complexity. Besides the use of sacrificial material to ensure good printability and softness upon removal, another strategy based on post-printing molecular cleavage was introduced by Wang et al*.*^[146]^ Although this method has only been applied to DLP, it may be used with VBP to fabricate constructs matching the mechanical properties of tissue.

Volumetric printing has only started to take advantage of the potential of photoclick reactions. Besides thiol-ene, several other photoclick reaction mechanisms, comprehensively reviewed elsewhere,^[^^98^^,^^99^^,137]^ remain currently unexplored. It is also worth mentioning that the library of possible photoclick reaction mechanisms goes beyond light-triggered free-radical mechanisms (reactions requiring a photoinitiator generating radical species), with photo-triggered uncaging,^[147]^ photodimerization,^[148–150]^ and photoregulated hydrazone/imine formation,^[151,152]^ presenting radical-free alternatives.

#### Silk-based hydrogels

Silkworm silk is a natural protein produced by the domesticated *Bombyx mori*; its main components include silk fibroin (SF) and silk sericin (SS), both of which are composed of 18 different amino acids but of very different ratios and properties.^[153–155]^ Reports of applying silk proteins, in particular SF, towards additive biomanufacturing, are abundant. These reports range from extrusion bioprinting where the biomaterial is used alone or in combination with other biomaterials as bioinks to support cellular functions^[156]^, or digital light processing bioprinting to exploit on-demand photocrosslinking to pattern cell-laden silk constructs in 3D.^[157]^ Photocrosslinking is usually achieved by functionalizing SF^[158–160]^ or SS^[160,161]^ with methacryloyl groups, followed by photoactivated structure-building in the presence of photoinitiators. More recently, it has been suggested that SF can also be used directly as a bioink in pristine form due to the development of the visible-light photoinitiator Ru/SPS that facilitates crosslinking through the formation of dityrosine bonds.^[162]^ For either extrusion bioprinting or stereolithography/digital light processing-enabled light-based bioprinting, the rapid formation of 3D silk constructs with sophisticated architectures usually requires protein concentrations > 5%.

**Figure 8:**
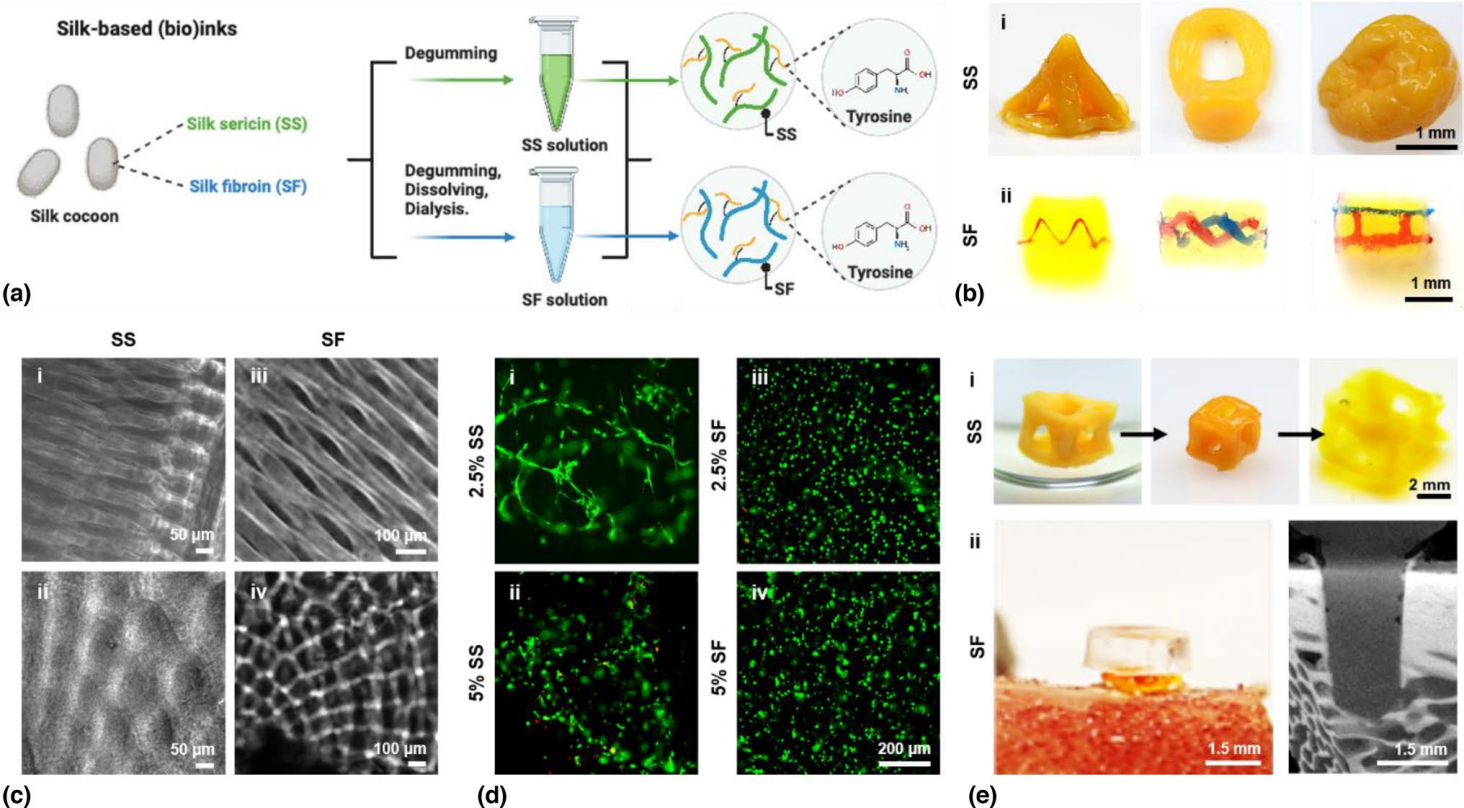
VBP of pristine silk hydrogels.^[18]^ (a) Silk-based (SS and SF) (bio)ink preparation and dy-tyrosine crosslinking. (b) (i) Photographs of VAM-printed SS objects. (ii) Photographs of VAM-printed SF objects. (c) Micrographs showing resolutions of VAM-printed (i, ii) SS and (iii, iv) SF patterns in the (i, iii) axial and (ii, iv) lateral directions. (d) Fluorescence micrographs showing C2C12 cell viability and spreading in VAM-bioprinted (i, ii) SS and (iii, iv) SF constructs of different protein concentrations, at day 14 of culture. Green: live; red: dead. (e) (i) Reversable shape-change for VAM-printed SS structure following immersion in 70% ethanol and rehydration in water; (ii) Photograph and micro-CT image showing ex vivo implantation of VAM-printed SF screw double-crosslinked by immersion in 70% ethanol in a porcine femur.

Inspired by the unique ability of tomographic volumetric additive manufacturing to decouple mechanical property requirements with the structural complexity attainable, as well as modification-free photocrosslinking enabled by the Ru/SPS photoinitiator system, the bioink pool for this bioprinting method was expanded to pristine silk biomaterials, including both SS and SF [Fig. 8(a)].^[18]^ Both unmodified SS and SF were volumetrically printed within a few tens of seconds to a few minutes. The printability range for SS was 2.5–5% SS paired with 0.25–1 mM of Ru (Ru/SPS ratio was kept at 1/10), while that for SF was slightly broader at 1.25–15% SF and 0.125–1 mM of Ru. Under optimized conditions, volumetric constructs containing sophisticated external shapes and internal architectures could be printed, such as a pyramid, ring, the brain-like structures for SS [Fig. 8(B-i)], and vascularized blocks for SF [Fig. 8(B-ii)]. Resolution assays further indicated the high resolution obtained with tomographic volumetric additive manufacturing of silk proteins. The axial resolution of printed SS reached 45.9 μm at minimum, while the smallest axial feature size of ~ 57 μm was attainable; in comparison, the lateral resolutions were generally at a poorer resolution, 108 μm for SS and 124 μm for SF [Fig. 8(c)].

Moreover, when cells were embedded in the inks, bioprinting of ultrasoft tissue constructs otherwise not easily obtained with other bioprinting strategies were achieved. When C2C12 myoblasts were encapsulated within the SS and SF bioinks for tomographic volumetric bioprinting, the cells showed high viability throughout 14 days of culture [Fig. 8(d)]. Interestingly, the cells within the bioprinted SS constructs at 2.5% exhibited significant spreading [Fig. 8(D-i)], indicating that the ultralow-concentration SS protein concentration facilitated cell growth better than the higher SS concentrations [Fig. 8(D-ii)], as well as SF at the same or higher concentrations [Fig. 8(D-iii, iv)]. A similar result was obtained with NIH/3T3 fibroblasts.

Finally, the tunable mechanical properties of the printed silk constructs further expanded the potential applications of these biomaterials towards biomedicine. The as-printed silk protein constructs, whether SS or SF, only contained photocrosslinked dityrosine bonds to form hydrogels. With secondary treatment post-printing, such as by immersing in ethanol, β-sheet content was induced, thus enhancing the mechanical properties of these hydrogels. For example, upon ethanol treatment, the compressive modulus of the printed 5% SS constructs increased from ~ 1 to ~ 40 kPa, associated with shrinkage in volume, while the shrinkage was reversible, where the structure could expand back to its original size and then swell to a larger size when immersed in water [Fig. 8(E-i)]. In contrast, the crosslinking was more extensive in the printed SF constructs upon ethanol treatment; compressive moduli were elevated from < 1 Kpa post-printing (dityrosine bonds only) to > 200 Mpa post-treatment (dityrosine bonds + β-sheets), also significantly higher than those for constructs crosslinked only with ethanol (β-sheets only). Thus, printed and double-crosslinked SF screws in the dry state were sufficiently robust that they could be used for fixing hard bone tissues [Fig. 8(E-ii)].

#### General perspectives for hydrogels and bioprinting

In recent years, the research field of 3D bioprinting and tissue engineering has seen a trend towards the use of high cell-density bioresins (tens, hundreds of million cells mL^−1^),^[163]^ which diverges from VBP current capabilities (≤ 15 million cells mL^−1^).^[20]^ Although the use of organoids,^[20]^ refractive index matching compounds,^[20]^ and optimized algorithms to limit light scattering^[34]^ have improved VBP performances, there is a major gap with more established methods such as extrusion printing or DLP.^[164]^ Near-infrared light could be used to increase penetration depth and alleviate the detrimental effects of scattering from high cell densities.

The precise 3D positioning (or patterning) of bioactive cues (i.e., small molecules, peptides and growth factors) within hydrogels has been traditionally obtained with lengthy two-photon triggered processes,^[165–169]^ VBP technology offers the possibility to rapidly distribute light doses in a 3D manner, thus triggering the patterning process in few seconds and for much larger volumes. The concept has been recently demonstrated by thiol-NB reactions,^[22]^ even to photograft complex patterns of growth factors and chemoattractants (i.e. vascular endothelial growth factor) while preserving their biological functionality,^[170]^ and is foreseen to become a major biological application of VBP in the near future.

As with every printing method, VBP comes with pros and cons, and while the technology is rapidly improving, a few groups have started to explore possible convergence with other printing methods, from melt electrowriting,^[23]^ to two photon ablation,^[144]^ and patterning.^[^^22^^,170]^ Table II lists examples of works which have integrated multiple fabrication methods with VAM/VBP and summarizes the demonstrated applications. Integrating fabrication methods leverages their strengths and mitigates their shortcomings. Exemplifying this, the work of Ribezzi et al*.*^[171]^ combines extrusion and volumetric bioprinting, which allow high cell density and the fabrication of hollow structures, respectively. The team used the two methods to fabricate densely islets laden with pancreatic β-cells that were extruded into a hydrogel laden with human mesenchymal stem cell and volumetrically bioprinted into a geometry mimicking pancreatic ducts.

In addition, VBP has so far mainly been used with single bioresins, therefore limiting the ability to resemble the complexity of human tissues that are made of different cell types and material compositions with regional distribution. Chansoria et al*.*, Groessbacher et al*.*, and Ribezzi et al*.* have demonstrated approaches for multimaterial and multicellular VBP, ^[^^23^^,145,171]^ opening new opportunities for VBP. Additionally, heterocellular structures have been fabricated through sequential VBP and cell injection in cartilage, bone and pancreatic models.^[10,21,45]^ Moreover, although not yet investigated in the presence of cells, the 3D stiffness gradients that can be obtained with localized consecutive exposures or dual wavelength exposures^[17]^ may add a pivotal level of biophysical complexity to the printed tissues.

Finally, from a material perspective, we believe that VBP will align with the most recent trends in the field and broaden its bioresins choice beyond gelatin, PEG and PVA with more biologically relevant systems such as collagen, decellularized ECM and viscoelastic networks featuring dynamic bonds.

### Supplementary Information

Below is the link to the electronic supplementary material.

Supplementary file1 (DOCX 35 kb).

## Data Availability

Data sharing is not applicable to this article as no new data were created or analyzed in this study.
