# Supplementary material for: A review of materials used in tomographic volumetric additive manufacturing
Source: MRS Commun. 2023 Aug 29;13(5):764–85. doi: 10.1557/s43579-023-00447-x (PMC10600040; doi:10.1557/s43579-023-00447-x)
Supplement: Supplementary file 1 — Supplementary file1 (DOCX 35 kb). [file 43579_2023_447_MOESM1_ESM.docx]

**A review of materials used in Tomographic Volumetric Additive Manufacturing**

Jorge Madrid-Wolff^1^, Joseph Toombs^2^, Riccardo Rizzo^3, 4^, Paulina Nuñez Bernal^5^, Dominique Porcincula^6^, Rebecca Walton^6^, Bin Wang^7^, Frederik Kotz-Helmer^8^, Yi Yang^9, 10^, David Kaplan^11^, Yu Shrike Zhang^12^, Marcy Zenobi-Wong^13^, Robert R. McLeod^14, 15^, Bastian Rapp^8^, Johanna Schwartz^6^, Maxim Shusteff^6^, Hayden Talyor^2^, Riccardo Levato^5,16^, Christophe Moser^1^

1. Ecole Polytechnique Féderale de Lausanne, CH-1015, Switzerland
2. Department of Mechanical Engineering, University of California, Berkeley, CA, USA
3. John A. Paulson School of Engineering and Applied Sciences, Harvard University, Cambridge, MA, USA
4. Wyss Institute for Biologically Inspired Engineering, Harvard University, Boston, MA, USA
5. Department of Orthopaedics, University Medical Center Utrecht, Utrecht University, the Netherlands
6. Lawrence Livermore National Laboratory, Livermore, CA, USA
7. Department of Mechanical Engineering, Technical University of Denmark, 2800, Kongens Lyngby, Denmark
8. Institute of Microstructure Technology (IMTEK), University of Freiburg, Georges Köhler Allee 103, 79110 Freiburg, Germany
9. Department of Chemistry, Technical University of Denmark (DTU), 2800 Kongens Lyngby, Denmark
10. Center for Energy Resources Engineering (CERE), Technical University of Denmark (DTU), 2800 Kongens Lyngby, Denmark
11. Department of Biomedical Engineering, Tufts University, Medford, MA, 02155, USA
12. Division of Engineering Medicine, Department of Medicine, Brigham and Women's Hospital, Harvard Medical School, Cambridge, MA 02139, USA
13. Tissue Engineering + Biofabrication Laboratory, Department of Health Sciences & Technology, ETH Zürich, Otto-Stern-Weg 7, Zürich, 8093 Switzerland
14. Materials Science and Engineering Program, University of Colorado, Boulder
15. Department of Electrical, Computer and Energy Engineering, University of Colorado, Boulder
16. Department of Clinical Sciences, Utrecht University, the Netherlands

**Supplementary References**

[101] C.-C. Lin, A. Raza, and H. Shih, “PEG hydrogels formed by thiol-ene photo-click chemistry and their effect on the formation and recovery of insulin-secreting cell spheroids,” Biomaterials, vol. 32, no. 36, pp. 9685–9695, Dec. 2011, doi: 10.1016/j.biomaterials.2011.08.083.

[102] S. B. Anderson, C.-C. Lin, D. V. Kuntzler, and K. S. Anseth, “The performance of human mesenchymal stem cells encapsulated in cell-degradable polymer-peptide hydrogels,” Biomaterials, vol. 32, no. 14, pp. 3564–3574, May 2011, doi: 10.1016/j.biomaterials.2011.01.064.

[103] L. A. Sawicki and A. M. Kloxin, “Design of thiol–ene photoclick hydrogels using facile techniques for cell culture applications,” Biomater. Sci., vol. 2, no. 11, pp. 1612–1626, Sep. 2014, doi: 10.1039/C4BM00187G.

[104] J. D. McCall and K. S. Anseth, “Thiol–Ene Photopolymerizations Provide a Facile Method To Encapsulate Proteins and Maintain Their Bioactivity,” Biomacromolecules, vol. 13, no. 8, pp. 2410–2417, Aug. 2012, doi: 10.1021/bm300671s.

[105] M. W. Tibbitt, A. M. Kloxin, L. A. Sawicki, and K. S. Anseth, “Mechanical Properties and Degradation of Chain and Step-Polymerized Photodegradable Hydrogels,” Macromolecules, vol. 46, no. 7, pp. 2785–2792, Apr. 2013, doi: 10.1021/ma302522x.

[106] A. F. Senyurt et al., “Physical and Mechanical Properties of Photopolymerized Thiol−Ene/Acrylates,” Macromolecules, vol. 39, no. 19, pp. 6315–6317, Sep. 2006, doi: 10.1021/ma060507f.

[107] N. B. Cramer, S. K. Reddy, A. K. O’Brien, and C. N. Bowman, “Thiol−Ene Photopolymerization Mechanism and Rate Limiting Step Changes for Various Vinyl Functional Group Chemistries,” Macromolecules, vol. 36, no. 21, pp. 7964–7969, Oct. 2003, doi: 10.1021/ma034667s.

[108] A. R. Kannurpatti, J. W. Anseth, and C. N. Bowman, “A study of the evolution of mechanical properties and structural heterogeneity of polymer networks formed by photopolymerizations of multifunctional (meth)acrylates,” Polymer, vol. 39, no. 12, pp. 2507–2513, Jan. 1998, doi: 10.1016/S0032-3861(97)00585-5.

[109] C. E. Hoyle and C. N. Bowman, “Thiol-Ene Click Chemistry,” Angewandte Chemie International Edition, vol. 49, no. 9, pp. 1540–1573, Feb. 2010, doi: 10.1002/anie.200903924.

[110] D. P. Nair et al., “Two-Stage Reactive Polymer Network Forming Systems,” Advanced Functional Materials, vol. 22, no. 7, Art. no. 7, 2012, doi: 10.1002/adfm.201102742.

[111] M. Podgórski et al., “Toward Stimuli‐Responsive Dynamic Thermosets through Continuous Development and Improvements in Covalent Adaptable Networks (CANs),” Adv. Mater., vol. 32, no. 20, p. 1906876, May 2020, doi: 10.1002/adma.201906876.

[112] D. P. Nair, N. B. Cramer, T. F. Scott, C. N. Bowman, and R. Shandas, “Photopolymerized thiol-ene systems as shape memory polymers,” Polymer, vol. 51, no. 19, Art. no. 19, Sep. 2010, doi: 10.1016/j.polymer.2010.07.027.

[113] J. Villacres, D. Nobes, and C. Ayranci, “Additive manufacturing of shape memory polymers: effects of print orientation and infill percentage on mechanical properties,” RPJ, vol. 24, no. 4, pp. 744–751, May 2018, doi: 10.1108/RPJ-03-2017-0043.

[114] J. Groll et al., “Biofabrication: reappraising the definition of an evolving field.,” Biofabrication, vol. 8, no. 1, p. 013001, 2016, doi: 10.1088/1758-5090/8/1/013001.

[115] V. Mironov, T. Trusk, V. Kasyanov, S. Little, R. Swaja, and R. Markwald, “Biofabrication: a 21st century manufacturing paradigm,” Biofabrication, vol. 1, no. 1, pp. 22001–16, 2009, doi: 10.1088/1758-5082/1/2/022001.

[116] J. Groll et al., “A definition of bioinks and their distinction from biomaterial inks,” Biofabrication, vol. 11, no. 1, 2019, doi: 10.1088/1758-5090/aaec52.

[117] R. Levato et al., “High-resolution lithographic biofabrication of hydrogels with complex microchannels from low-temperature-soluble gelatin bioresins,” Materials Today Bio, 2021, doi: 10.1016/j.mtbio.2021.100162.

[118] R. Levato, T. Jungst, R. G. Scheuring, T. Blunk, J. Groll, and J. Malda, “From Shape to Function: The Next Step in Bioprinting,” Advanced Materials, vol. 32, no. 12, 2020, doi: 10.1002/adma.201906423.

[119] H. W. Kang, S. J. Lee, I. K. Ko, C. Kengla, J. J. Yoo, and A. Atala, “A 3D bioprinting system to produce human-scale tissue constructs with structural integrity,” Nature Biotechnology, vol. 34, no. 3, pp. 312–319, 2016, doi: 10.1038/nbt.3413.

[120] M. A. Heinrich, R. Bansal, T. Lammers, Y. S. Zhang, R. Michel Schiffelers, and J. Prakash, “3D-Bioprinted Mini-Brain: A Glioblastoma Model to Study Cellular Interactions and Therapeutics,” Advanced Materials, vol. 31, no. 14, 2019, doi: 10.1002/adma.201806590.

[121] A. Lode et al., “Green bioprinting: Fabrication of photosynthetic algae-laden hydrogel scaffolds for biotechnological and medical applications,” Engineering in Life Sciences, vol. 15, no. 2, pp. 177–183, 2015, doi: 10.1002/elsc.201400205.

[122] T. Zandrini, S. Florczak, R. Levato, and A. Ovsianikov, “Breaking the resolution limits of 3D bioprinting: future opportunities and present challenges,” Trends in Biotechnology, 2022, doi: 10.1016/j.tibtech.2022.10.009.

[123] A. I. Van Den Bulcke, B. Bogdanov, N. De Rooze, E. H. Schacht, M. Cornelissen, and H. Berghmans, “Structural and Rheological Properties of Methacrylamide Modified Gelatin Hydrogels,” Biomacromolecules, vol. 1, no. 1, pp. 31–38, Mar. 2000, doi: 10.1021/bm990017d.

[124] D. Loessner et al., “Functionalization, preparation and use of cell-laden gelatin methacryloyl-based hydrogels as modular tissue culture platforms.,” Nature protocols, vol. 11, no. 4, pp. 727–46, 2016, doi: 10.1038/nprot.2016.037.

[125] H. Shirahama, B. H. Lee, L. P. Tan, and N.-J. Cho, “Precise Tuning of Facile One-Pot Gelatin Methacryloyl (GelMA) Synthesis,” Sci Rep, vol. 6, no. 1, Art. no. 1, Aug. 2016, doi: 10.1038/srep31036.

[126] W. M. G. A. C. Groen et al., “Impact of endotoxins in gelatine hydrogels on chondrogenic differentiation and inflammatory cytokine secretion in vitro,” International Journal of Molecular Sciences, 2020, doi: 10.3390/ijms21228571.

[127] T. Billiet, E. Gevaert, T. De Schryver, M. Cornelissen, and P. Dubruel, “The 3D printing of gelatin methacrylamide cell-laden tissue-engineered constructs with high cell viability,” Biomaterials, vol. 35, no. 1, pp. 49–62, 2014, doi: 10.1016/j.biomaterials.2013.09.078.

[128] R. Levato, J. Visser, J. a Planell, E. Engel, J. Malda, and M. a Mateos-Timoneda, “Biofabrication of tissue constructs by 3D bioprinting of cell-laden microcarriers.,” Biofabrication, vol. 6, no. 3, p. 035020, 2014, doi: 10.1088/1758-5082/6/3/035020.

[129] M. Costantini et al., “3D bioprinting of BM-MSCs-loaded ECM biomimetic hydrogels for in vitro neocartilage formation,” Biofabrication, vol. 8, no. 3, p. 035002, Jul. 2016, doi: 10.1088/1758-5090/8/3/035002.

[130] R. Gauvin et al., “Microfabrication of complex porous tissue engineering scaffolds using 3D projection stereolithography,” Biomaterials, vol. 33, no. 15, pp. 3824–3834, 2012, doi: 10.1016/j.biomaterials.2012.01.048.

[131] M. C. Bouwmeester et al., “Bioprinting of Human Liver-Derived Epithelial Organoids for Toxicity Studies,” Macromolecular Bioscience, 2021, doi: 10.1002/mabi.202100327.

[132] T. Boothe et al., “A tunable refractive index matching medium for live imaging cells, tissues and model organisms,” eLife, vol. 6, p. e27240, Jul. 2017, doi: 10.7554/eLife.27240.

[133] B. J. Klotz et al., “Engineering of a complex bone tissue model with endothelialised channels and capillary-like networks,” European Cells and Materials, vol. 35, pp. 335–349, 2018, doi: 10.22203/eCM.v035a23.

[134] K. S. Lim et al., “Visible light cross-linking of gelatin hydrogels offers an enhanced cell microenvironment with improved light penetration depth,” Macromolecular Bioscience, vol. 19, no. 6, p. 1900098, 2019.

[135] B. G. Soliman et al., “Development and Characterization of Gelatin-Norbornene Bioink to Understand the Interplay between Physical Architecture and Micro-Capillary Formation in Biofabricated Vascularized Constructs,” Advanced Healthcare Materials, vol. 11, no. 2, p. 2101873, 2022, doi: 10.1002/adhm.202101873.

[136] K. S. Lim et al., “One-Step Photoactivation of a Dual-Functionalized Bioink as Cell Carrier and Cartilage-Binding Glue for Chondral Regeneration,” Advanced Healthcare Materials, vol. 9, no. 15, 2020, doi: 10.1002/adhm.201901792.

[137] M. Lee, R. Rizzo, F. Surman, and M. Zenobi-Wong, “Guiding Lights: Tissue Bioprinting Using Photoactivated Materials,” Chem. Rev., vol. 120, no. 19, pp. 10950–11027, Oct. 2020, doi: 10.1021/acs.chemrev.0c00077.

[138] C. E. Hoyle, T. Y. Lee, and T. Roper, “Thiol–enes: Chemistry of the past with promise for the future,” Journal of Polymer Science Part A: Polymer Chemistry, vol. 42, no. 21, pp. 5301–5338, 2004, doi: 10.1002/pola.20366.

[139] E. R. Ruskowitz and C. A. DeForest, “Proteome-wide Analysis of Cellular Response to Ultraviolet Light for Biomaterial Synthesis and Modification,” ACS Biomater. Sci. Eng., vol. 5, no. 5, pp. 2111–2116, May 2019, doi: 10.1021/acsbiomaterials.9b00177.

[140] M. Y. Kwon, C. Wang, J. H. Galarraga, E. Puré, L. Han, and J. A. Burdick, “Influence of hyaluronic acid modification on CD44 binding towards the design of hydrogel biomaterials,” Biomaterials, vol. 222, p. 119451, Nov. 2019, doi: 10.1016/j.biomaterials.2019.119451.

[141] S. J. Bryant, T. T. Chowdhury, D. A. Lee, D. L. Bader, and K. S. Anseth, “Crosslinking Density Influences Chondrocyte Metabolism in Dynamically Loaded Photocrosslinked Poly(ethylene glycol) Hydrogels,” Annals of Biomedical Engineering, vol. 32, no. 3, pp. 407–417, Mar. 2004, doi: 10.1023/B:ABME.0000017535.00602.ca.

[142] L. Bian, C. Hou, E. Tous, R. Rai, R. L. Mauck, and J. A. Burdick, “The influence of hyaluronic acid hydrogel crosslinking density and macromolecular diffusivity on human MSC chondrogenesis and hypertrophy,” Biomaterials, vol. 34, no. 2, pp. 413–421, Jan. 2013, doi: 10.1016/j.biomaterials.2012.09.052.

[143] K. Wolf et al., “Physical limits of cell migration: control by ECM space and nuclear deformation and tuning by proteolysis and traction force,” J Cell Biol, vol. 201, no. 7, pp. 1069–1084, Jun. 2013, doi: 10.1083/jcb.201210152.

[144] R. Rizzo et al., “Multiscale Hybrid Fabrication: Volumetric Printing Meets Two-Photon Ablation,” Advanced Materials Technologies, p. 2201871, Mar. 2023, doi: 10.1002/admt.202201871.

[145] P. Chansoria et al., “Synergizing algorithmic design, photoclick chemistry and multi-material volumetric printing for accelerating complex shape engineering.” bioRxiv, p. 2022.11.29.518318, Dec. 02, 2022. doi: 10.1101/2022.11.29.518318.

[146] M. Wang et al., “Molecularly cleavable bioinks facilitate high-performance digital light processing-based bioprinting of functional volumetric soft tissues,” Nat Commun, vol. 13, no. 1, Art. no. 1, Jun. 2022, doi: 10.1038/s41467-022-31002-2.

[147] R. Rizzo, N. Petelinšek, A. Bonato, and M. Zenobi-Wong, “From Free-Radical to Radical-Free: A Paradigm Shift in Light-Mediated Biofabrication,” Advanced Science, vol. 10, no. 8, p. 2205302, 2023, doi: 10.1002/advs.202205302.

[148] V. X. Truong, F. Li, and J. S. Forsythe, “Versatile Bioorthogonal Hydrogel Platform by Catalyst-Free Visible Light Initiated Photodimerization of Anthracene,” ACS Macro Lett., vol. 6, no. 7, pp. 657–662, Jul. 2017, doi: 10.1021/acsmacrolett.7b00312.

[149] R. Beninatto et al., “Photocrosslinked hydrogels from coumarin derivatives of hyaluronic acid for tissue engineering applications,” Materials Science and Engineering: C, vol. 96, pp. 625–634, Mar. 2019, doi: 10.1016/j.msec.2018.11.052.

[150] R. Tamate et al., “Photocurable ABA triblock copolymer-based ion gels utilizing photodimerization of coumarin,” RSC Adv., vol. 8, no. 7, pp. 3418–3422, Jan. 2018, doi: 10.1039/C7RA13181J.

[151] M. A. Azagarsamy, I. A. Marozas, S. Spaans, and K. S. Anseth, “Photoregulated Hydrazone-Based Hydrogel Formation for Biochemically Patterning 3D Cellular Microenvironments,” ACS Macro Lett., vol. 5, no. 1, pp. 19–23, Jan. 2016, doi: 10.1021/acsmacrolett.5b00682.

[152] Y. Yang et al., “Tissue-Integratable and Biocompatible Photogelation by the Imine Crosslinking Reaction,” Advanced Materials, vol. 28, no. 14, pp. 2724–2730, 2016, doi: 10.1002/adma.201505336.

[153] H. Tao, D. L. Kaplan, and F. G. Omenetto, “Silk Materials – A Road to Sustainable High Technology,” Advanced Materials, vol. 24, no. 21, pp. 2824–2837, 2012, doi: 10.1002/adma.201104477.

[154] D. Ebrahimi, O. Tokareva, N. G. Rim, J. Y. Wong, D. L. Kaplan, and M. J. Buehler, “Silk–Its Mysteries, How It Is Made, and How It Is Used,” ACS Biomater. Sci. Eng., vol. 1, no. 10, pp. 864–876, Oct. 2015, doi: 10.1021/acsbiomaterials.5b00152.

[155] C. Guo, C. Li, X. Mu, and D. L. Kaplan, “Engineering silk materials: From natural spinning to artificial processing,” Applied Physics Reviews, vol. 7, no. 1, p. 011313, Mar. 2020, doi: 10.1063/1.5091442.

[156] S. Chawla, S. Midha, A. Sharma, and S. Ghosh, “Silk-Based Bioinks for 3D Bioprinting,” Advanced Healthcare Materials, vol. 7, no. 8, p. 1701204, 2018, doi: 10.1002/adhm.201701204.

[157] S. H. Kim, D. Y. Kim, T. H. Lim, and C. H. Park, “Silk Fibroin Bioinks for Digital Light Processing (DLP) 3D Bioprinting,” in Bioinspired Biomaterials: Advances in Tissue Engineering and Regenerative Medicine, H. J. Chun, R. L. Reis, A. Motta, and G. Khang, Eds., in Advances in Experimental Medicine and Biology. Singapore: Springer, 2020, pp. 53–66. doi: 10.1007/978-981-15-3258-0_4.

[158] S. H. Kim et al., “Precisely printable and biocompatible silk fibroin bioink for digital light processing 3D printing,” Nat Commun, vol. 9, no. 1, Art. no. 1, Apr. 2018, doi: 10.1038/s41467-018-03759-y.

[159] S. H. Kim et al., “3D bioprinted silk fibroin hydrogels for tissue engineering,” Nat Protoc, vol. 16, no. 12, Art. no. 12, Dec. 2021, doi: 10.1038/s41596-021-00622-1.

[160] J.-X. Li, S.-X. Zhao, and Y.-Q. Zhang, “Silk Protein Composite Bioinks and Their 3D Scaffolds and In Vitro Characterization,” International Journal of Molecular Sciences, vol. 23, no. 2, Art. no. 2, Jan. 2022, doi: 10.3390/ijms23020910.

[161] S. Kader and E. Jabbari, “Material Properties and Cell Compatibility of Photo-Crosslinked Sericin Urethane Methacryloyl Hydrogel,” Gels, vol. 8, no. 9, Art. no. 9, Sep. 2022, doi: 10.3390/gels8090543.

[162] X. Cui et al., “Rapid Photocrosslinking of Silk Hydrogels with High Cell Density and Enhanced Shape Fidelity,” Advanced Healthcare Materials, vol. 9, no. 4, p. 1901667, 2020, doi: 10.1002/adhm.201901667.

[163] K. J. Wolf, J. D. Weiss, S. G. M. Uzel, M. A. Skylar-Scott, and J. A. Lewis, “Biomanufacturing human tissues via organ building blocks,” Cell Stem Cell, vol. 29, no. 5, pp. 667–677, May 2022, doi: 10.1016/j.stem.2022.04.012.

[164] S. You et al., “High cell density and high-resolution 3D bioprinting for fabricating vascularized tissues,” Science Advances, vol. 9, no. 8, p. eade7923, Feb. 2023, doi: 10.1126/sciadv.ade7923.

[165] C. A. DeForest and K. S. Anseth, “Photoreversible Patterning of Biomolecules within Click-Based Hydrogels,” Angewandte Chemie International Edition, vol. 51, no. 8, pp. 1816–1819, 2012, doi: 10.1002/anie.201106463.

[166] N. Broguiere et al., “Morphogenesis Guided by 3D Patterning of Growth Factors in Biological Matrices,” Advanced Materials, vol. 32, no. 25, p. 1908299, 2020, doi: 10.1002/adma.201908299.

[167] H. Krüger, M. Asido, J. Wachtveitl, R. Tampé, and R. Wieneke, “Sensitizer-enhanced two-photon patterning of biomolecules in photoinstructive hydrogels,” Commun Mater, vol. 3, no. 1, Art. no. 1, Feb. 2022, doi: 10.1038/s43246-022-00230-w.

[168] Y. Aizawa, R. Wylie, and M. Shoichet, “Endothelial Cell Guidance in 3D Patterned Scaffolds,” Advanced Materials, vol. 22, no. 43, pp. 4831–4835, 2010, doi: 10.1002/adma.201001855.

[169] X.-H. Qin, X. Wang, M. Rottmar, B. J. Nelson, and K. Maniura-Weber, “Near-Infrared Light-Sensitive Polyvinyl Alcohol Hydrogel Photoresist for Spatiotemporal Control of Cell-Instructive 3D Microenvironments,” Advanced Materials, vol. 30, no. 10, p. 1705564, 2018, doi: 10.1002/adma.201705564.

[170] M. Falandt et al., “Spatial-Selective Volumetric 4D Printing and Single-Photon Grafting of Biomolecules within Centimeter-Scale Hydrogels via Tomographic Manufacturing,” Advanced Materials Technologies, vol. n/a, no. n/a, p. 2300026, doi: 10.1002/admt.202300026.

[171] D. Ribezzi et al., “Shaping Synthetic Multicellular and Complex Multimaterial Tissues via Embedded Extrusion-Volumetric Printing of Microgels,” Advanced Materials, vol. n/a, no. n/a, p. 2301673, doi: 10.1002/adma.202301673.
